# Best Practicable Aggregation of Species: a step forward for species surrogacy in environmental assessment and monitoring

**DOI:** 10.1002/ece3.715

**Published:** 2013-09-11

**Authors:** Stanislao Bevilacqua, Joachim Claudet, Antonio Terlizzi

**Affiliations:** 1 Laboratory of Zoology and Marine Biology, Department of Biological and Environmental Sciences and Technologies, University of Salento73100, Lecce, Italy; 2 National Center for Scientific Research, USR 3278 CNRS-EPHE CRIOBE, University of Perpignan66860, Perpignan Cedex, France; 3 Laboratoire d'Excellence ‘CORAIL’, USR 3278 CNRS-EPHE CRIOBE, University of Perpignan66860, Perpignan Cedex, France

**Keywords:** Environmental impacts, higher taxon approach, modeling, multivariate analysis, natural variations, randomizations, species surrogates, taxonomic relatedness, taxonomic sufficiency.

## Abstract

The available taxonomic expertise and knowledge of species is still inadequate to cope with the urgent need for cost-effective methods to quantifying community response to natural and anthropogenic drivers of change. So far, the mainstream approach to overcome these impediments has focused on using higher taxa as surrogates for species. However, the use of such taxonomic surrogates often limits inferences about the causality of community patterns, which in turn is essential for effective environmental management strategies. Here, we propose an alternative approach to species surrogacy, the “Best Practicable Aggregation of Species” (BestAgg), in which surrogates exulate from fixed taxonomic schemes. The approach uses null models from random aggregations of species to minimizing the number of surrogates without causing significant losses of information on community patterns. Surrogate types are then selected in order to maximize ecological information. We applied the approach to real case studies on natural and human-driven gradients from marine benthic communities. Outcomes from BestAgg were also compared with those obtained using classic taxonomic surrogates. Results showed that BestAgg surrogates are effective in detecting community changes. In contrast to classic taxonomic surrogates, BestAgg surrogates allow retaining significantly higher information on species-level community patterns than what is expected to occur by chance and a potential time saving during sample processing up to 25% higher. Our findings showed that BestAgg surrogates from a pilot study could be used successfully in similar environmental investigations in the same area, or for subsequent long-term monitoring programs. BestAgg is virtually applicable to any environmental context, allowing exploiting multiple surrogacy schemes beyond stagnant perspectives strictly relying on taxonomic relatedness among species. This prerogative is crucial to extend the concept of species surrogacy to ecological traits of species, thus leading to ecologically meaningful surrogates that, while cost effective in reflecting community patterns, may also contribute to unveil underlying processes. A specific R code for BestAgg is provided.

## Introduction

The unprecedented increase in anthropogenic disturbance worldwide has exacerbated concerns about the potential ensuing depletion of biodiversity and ecosystem functioning (Hooper et al. 2012). However, the intrinsic complexity of ecological systems largely limits our ability to predict their possible critical transitions toward undesirable states (Scheffer et al. 2012). Environmental impact assessment and monitoring, therefore, are of basic importance in revealing the effects of human pressures and their interactions with natural sources of variability, detecting early signals of phase shifts, and guiding subsequent adaptive management and mitigation strategies (Hill and Arnold 2012).

Wide gaps in knowledge of phylogenetic, taxonomic, and functional characteristics of most species (Lomolino 2004; Whittaker et al. 2005; Cardoso et al. 2011) make difficult quantifying human-driven patterns of changes and unveiling underlying ecological processes. Progresses in molecular analyses, such as DNA bar coding of organisms, are helping the process of cataloging biodiversity (Gross 2012), and recent developments in this field highlighted the value of genetic tagging in estimating ecological properties of communities despite the inherent loss of taxonomic information (e.g., Fonseca et al. 2010; Yu et al. 2012). Molecular analyses and bioinformatics, nevertheless, represent complementary but not alternative approaches to huge endeavors for research in taxonomy and autoecology (Wilson 2004), which are inevitable for advancing the knowledge of biodiversity (May 1990; Wheeler 2004; Wheeler et al. 2004; de Carvalho et al. 2007).

Despite renewed efforts in the exploration of biodiversity (e.g., Snelgrove 2010; Fontaine et al. 2012) and in the enhancement of taxonomy and systematics (Boero 2001; Wilson 2003), current knowledge of species is still far from being exhaustive (Pereira et al. 2012) and the availability of taxonomic expertise appears still insufficient (Wägele et al. 2011) to cope with the current need of timely solutions to pressing environmental problems.

This so-called ‘taxonomic impediment’ (e.g., Wheeler 2004) is challenging in applied ecological research to provide cost-effective methods for elucidating the response of communities and ecosystems to natural and anthropogenic drivers of change (Pik et al. 1999; Jones 2008; Mandelik et al. 2010; Mellin et al. 2011). A mainstream practice to overcome this hindrance across terrestrial, freshwater, and marine environments focuses on the use of higher taxa as surrogates for species (Bevilacqua et al. 2012). The higher taxon approach in environmental investigations is based on the concept of taxonomic sufficiency, which involves the use of coarse taxonomic resolution without causing a significant loss of information, thus avoiding costly, time-expensive, and difficult species-level identifications (Beattie and Oliver 1994). Such an approach, especially when based on intermediate taxonomic ranks (i.e., Genus and Family), is generally effective in depicting species-level patterns of community response under a wide range of environmental settings (e.g., Heino and Soininen 2007; Lovell et al. 2007; Terlizzi et al. 2009).

However, taxonomic sufficiency implies the static grouping of organisms in taxa belonging to a single taxonomic level higher than species (e.g., all organisms identified as genera, or families, etc.) irrespective of their ecological relevance or difficulty of taxonomic identifications. As a consequence, the use of higher taxa as surrogates for species (hereafter referred to as taxonomic surrogates) often restricts inferences about the causality of the observed patterns (Lenat and Resh 2001; Terlizzi et al. 2003; Jones 2008).

Uncertainties about the appropriateness of this approach to species surrogacy may depend on the fact that related empirical studies have amassed in the absence of incisive efforts in structuring a solid theoretical framework for the application of taxonomic surrogates. Putative similarities in ecological traits among closely related species, or hierarchical (from species to higher taxonomic ranks) responses to environmental disturbance, have been invoked to substantiate the ability of taxonomic surrogates to mirror species-level patterns (e.g., Warwick 1993; Ferraro and Cole 1990; Heino and Soininen 2007). Such explanations are, nevertheless, unable to elucidate exhaustively the reasons behind the success, or failure, of taxonomic surrogates (Lenat and Resh 2001; Bertrand et al. 2006; Dethier and Schoch 2006; Bevilacqua et al. 2009), and are difficult to validate experimentally. The absence of clearly stated assumptions on the effectiveness of taxonomic surrogates, and the lack of standard methods for quantifying the probability of Type-I error when identifying a particular taxonomic level as effective in discerning a given pattern of interest, raised criticism about their potential utility (Mellin et al. 2011).

Several studies that have investigated factors affecting the performance of taxonomic surrogates, such as taxonomic relatedness among species, outlined that higher taxa perform better as surrogates for species when they are poor in species (e.g., Lovell et al. 2007), or there is a small mean and variance in the number of species per higher taxon (e.g., Neeson et al. 2013) or, in other words, when the ratio of the number of species to the number of higher taxa is low (e.g., Giangrande et al. 2005; Dethier and Schoch 2006). In a recent attempt to shed light on potential mechanisms determining the performance of taxonomic surrogates, Bevilacqua et al. (2012), working on marine molluscs at a regional scale, used null models to show that higher taxa of the Linnaean taxonomic hierarchy may be considered as arbitrary categories of species unlikely to convey consistent responses to natural or human-driven environmental changes. A similar approach, based on the metacommunity concept, led Siqueira et al. (2012) to analogous conclusions when investigating congruences in spatial patterns of variation in community composition of freshwater invertebrates among the whole set and different subset taxa. Bevilacqua et al. (2012) showed that information loss and the ensuing decrease in statistical power to detect changes in assemblage structure at higher taxonomic levels depended on the degree of species aggregation (exemplified by the ratio between the number of higher taxa and the number of species), rather than on taxonomic relatedness of species (i.e., the relative closeness of species in the Linnaean taxonomic hierarchy) (see also Siqueira et al. 2012 for similar findings). By analyzing 20 years of research on taxonomic surrogates, the authors also found strong evidence supporting the generality of such findings across a wide range of terrestrial, freshwater, and marine organisms.

In this perspective, here, we propose a novel approach to species surrogacy, the Best Practicable Aggregation of Species (BestAgg), that allows alternative ways to aggregate species into surrogates, beyond static taxonomic grouping, in order to maximizing ecological information and to optimizing the use of surrogates for species in ecological studies. Taxonomic sufficiency concerns the use of higher taxa as surrogates for species and aims to identifying the coarser level of taxonomic resolution sufficient to allow the assessment of community response to environmental drivers. The BestAgg approach, instead, relies on determining the sufficient (i.e., minimum) number of surrogate groups, irrespective of their type (i.e., if taxonomic, morphological, functional, etc.), that could be used while still obtaining consistent results with species-level community response. As for any rigorous surrogacy approach (e.g., Van Wynsberge et al. 2012), taxonomic sufficiency relies on a first assessment of the sufficient taxonomic resolution based on species-level data (e.g., Terlizzi et al. 2003; Defeo and Lercari 2004; Jones 2008). In this framework, a pilot investigation compares results of analyses at species level with those obtained using higher taxa. Species-level data are therefore aggregated (i.e., grouped and summed) into higher taxa and the coarser taxonomic resolution able to provide consistent results with those obtained from species-level data is assumed to be suitable for subsequent monitoring or for very similar study contexts.

Following the same framework, we used species-level information from pilot studies to identify the sufficient number of surrogates able to depict community patterns consistently with species-level information. Surrogates were then defined based on their ecological importance (*relevance*), low difficulty of taxonomic identification during sample processing (*easiness*), and shared characteristics among organisms (*resemblance*). Finally, we tested the performance of BestAgg surrogates in similar study contexts and compared their response with classic surrogates based on taxonomy (i.e., higher taxa).

## Methods

### The BestAgg: assumptions and rationale

The identification of the sufficient number of surrogates is based on a null model assuming that surrogates may be considered as random groups of species from the original species pool found in the study (see Bevilacqua et al. 2012 for a full theoretical discussion). Specifically, the model assumes that (1) the ability of surrogates to exhibit the same community response detectable at species level depends on the residual information retained in the aggregated data. This residual information can be expressed as the Spearman's correlation (ρ) between the species-level data matrix and the corresponding aggregated matrix (Somerfield and Clarke 1995). The bulk of evidence on the application of taxonomic surrogates widely supports this assumption (Bevilacqua et al. 2012).

However, while for taxonomic sufficiency the coherence in community responses between species and higher taxonomic ranks would originate from the putative ecological similarities among species within higher taxa (e.g., Warwick 1993), the rationale underlying BestAgg is that it is the level of aggregation that matters or, in other words, the number of surrogates in which species are aggregated, irrespective of underlying aggregation criteria. In this view, it is assumed that (2) the residual information in the aggregated matrix (ρ) depends on the level of aggregation, which can be expressed as ϕ = *G*/*S*, that is, the ratio of the number of surrogates groups *G* in which species are aggregated to the number of species *S* in the original species-level matrix. Such a dependence between ρ and ϕ has been largely confirmed analyzing the scientific literature on taxonomic surrogates, which showed that the decrease in ρ at decreasing ϕ follows a semilog model consistent across different organisms and habitat types (Bevilacqua et al. 2012). Under the two aforementioned assumptions, the effectiveness of a given set of surrogates for species (i.e., their ability to emulate species-level community response) depends on the level of aggregation ϕ.

The BestAgg approach consists of two main steps. First, a null model based on random aggregations of species-level data is built to determine the value of ϕ, namely, ϕ_low_ (i.e., the lowest practicable aggregation level), sufficient to obtain results consistent with those obtained analyzing species-level data. Second, the study-specific surrogate groups are identified on the basis of ϕ_low_ and a set of selection criteria aiming to capitalize on the ecological information in the aggregated matrix.

### Null model based on randomly aggregated matrices

Let *M*_*S*_ be a matrix *S* × *N* of any type of abundance data collected at species level from any assemblage, where *S* is the number of species and *N* the number of samples. The aggregated matrix derived from *M*_*S*_ is a *G* × *N* matrix in which all the original *S* species have been assigned to *G* groups (with *G* < *S*) and their abundance summed. The level of aggregation of this matrix is defined as ϕ = *G*/*S*.

**Figure 1:**
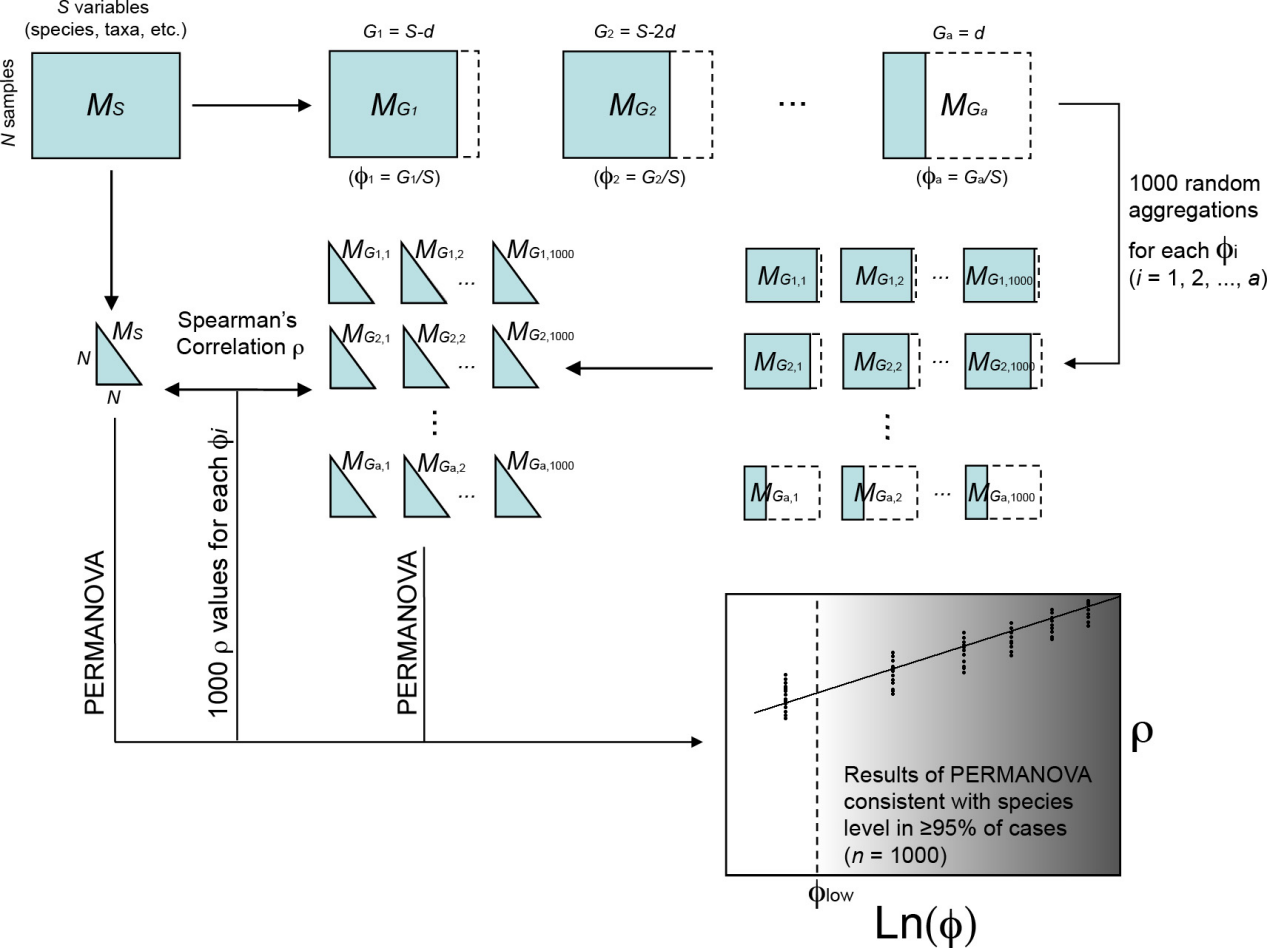
Construction of the null model for the identification of the lowest practicable level of aggregation (ϕ_low_) in BestAgg. The *S* variables (e.g., species, taxa, groups, etc.) in the original *S* × *N* matrix (*M*_*S*_) are aggregated (i.e., grouped and summed) at random into a progressively decreasing number of *G*_*i*_ variables following a stepwise reduction in fixed percentage detriments *d* (details in the text). For each *G*_*i*_, random aggregations of the original *S* variables are repeated, obtaining 1000 randomly aggregated *G*_*i*_ × *S* matrices (*M*_*G*__*i*_) with a level of aggregation ϕ_*i*_. The level of aggregation for a given matrix is defined as ϕ_*i*_ = *G*_*i*_/*S*. For each randomly aggregated matrix and for the original matrix (i.e., *M*_*S*_), the corresponding triangular matrix based on any distance measure of choice is obtained. A distance-based multivariate analysis of variance (PERMANOVA) (see text for further details) is performed based on all triangular matrices (the original one and all the derived randomly aggregated matrices) to test for the factor of interest in the experiment. The Spearman's correlation ρ between the triangular *M*_*S*_ and each of the 1000 triangular *M*_*G*__*i*_ is also calculated. Finally, a semilog model of ρ against ln(ϕ) based on results is fitted in order to identify the lowest ϕ value at which ≥95% of PERMANOVA tests on randomly aggregated matrices provides results consistent with those obtained analyzing the original data matrix (i.e., ϕ_low_).

The whole procedure for the construction of the null model is synthesized in Figure 1. The aim of the approach is to identify the lowest ϕ value at which (1) there is consistently a strong correlation between the species and surrogate dissimilarity matrices, regardless of how species are aggregated into surrogate groups, and (2) the probability of statistical tests to fail in detecting significant differences for the term of interest in the analysis when using the corresponding number of surrogates is *P* < 0.05.

For the construction of the null model, the *S* species in the original matrix *M*_*S*_ are randomly aggregated in a decreasing number of *G* groups in order to simulate decreasing values of ϕ. The number of groups *G* is progressively decreased, starting from *G* = *S*, by stepwise reductions of a fixed decrement *d*. Decrements of *d* = 10^−1^ × *S* (i.e., 10% of *S*) allow defining a representative set of simulated decreasing ϕ values for a wide range of *S*, and are therefore suitable in most studies concerning species surrogacy (Bevilacqua et al. 2012). If necessary, that is, in the analyses of speciose assemblages, decrements can even be set to 5% of *S* to avoid excessive gaps in the progressive reduction in *G*.

For each set of *G* groups obtained from the stepwise reduction procedure, random aggregations are repeated 1000 times, obtaining 1000 random aggregated matrices for each corresponding value of ϕ. A triangular matrix based on any measure of resemblance (e.g., Jaccard, Sørensen, Bray–Curtis, etc.) can be obtained from the original species-level matrix *M*_*S*_ and from each aggregated matrix. Then, for each aggregated triangular matrix, the correlation value ρ with the triangular *M*_*s*_ is calculated. Next, a distance-based permutational multivariate analysis of variance (PERMANOVA, Anderson 2001) is performed based on each randomly aggregated matrix to test for the term of interest in the analysis (e.g., significant effects of the investigated factor, such as a natural or human-induced environmental gradient, on multivariate assemblage structure). PERMANOVA has been used as default multivariate statistical test because it allows analyzing complex multifactorial designs and testing for interaction terms (in contrast to, for instance, analysis of similarities (ANOSIM), which allows analyzing only two-factor designs and does not test for interactions) (ANOSIM, see Clarke 1993), using any distance measure and overcoming problems related to non-normality of data (in contrast to, for instance, classic MANOVA, which requires normality and implicitly uses Euclidean distance) (see Legendre and Anderson 1999; Anderson 2001; McArdle and Anderson 2001).

The dependence between ϕ and the effectiveness of the corresponding surrogate groups, which is the logic derivation of model assumptions (1) and (2) (see previous Methods section), can be now formally checked by fitting a semilog model of ρ values against the corresponding ϕ values and calculating, for each level of aggregation ϕ, the percentage of significant PERMANOVA tests for the term of interest, out of *n* = 1000, consistent with results obtained at species level. Significant results from analyses on aggregated data are considered as consistent with those obtained at species level if *α*_*G*_ ≤ *α*_*S*_, where *α*_*S*_ is the significance level of the test for the term of interest (i.e., *α*_*S*_ = 0.05, if 0.01 < *P* < 0.05; *α*_*S*_ = 0.01, if 0.001 < *P* < 0.01; *α*_*S*_ = 0.001, if *P* < 0.001) based on species-level data, and *α*_*G*_ is the significance level of the same test from the analysis on aggregated data.

Finally, the lowest practicable aggregation level ϕ_low_ is determined by identifying the lowest ϕ value allowing the 95% of PERMANOVAs on randomly aggregated matrices to give results consistent with those obtained at species level. Given that ϕ = *G*/*S*, it is possible to derive the sufficient (or, in other words, the *minimum*) number of surrogate groups *G*_min_ = ϕ_low_ × *S* needed to obtain consistent results to those obtained analyzing species-level data. In such cases we can reject the null hypothesis that *G*_min_ is not sufficient to allow consistent results with species-level analysis with a probability of Type-I error of *P* < 0.05, under the assumption that surrogate groups are random subsets of species. It is worth noting here that *G*_min_ represents a threshold value. The number of surrogate groups that could be employed is not required to be necessarily equal to *G*_min_, but just to be within the range of sufficient aggregation levels for analyses or, in other words, ≥*G*_min_.

The R code for analyses along with a brief user manual is provided (see Appendices S1 and S2). Example data are also supplied (see Appendices S3 and S4).

### Selecting surrogate groups for BestAgg

Once *G*_min_ is set, the subsequent step concerns the selection of surrogates. Formally, it could be virtually criterion free, as species could be grouped randomly within the *G*_min_ surrogate groups. However, the approach does not intend to legitimate random aggregations of species, but, rather, to achieve an aggregation of species coherent with the aim of the study and the knowledge of the investigated system.

The concept underlying BestAgg is to exploit simultaneously the potential of different surrogate types in providing cost-effective assessments of community response. The approach, while fixing the sufficient number of surrogates that could be used, unleash the investigator from static surrogacy schemes, allowing the selection of any surrogate for species potentially leading to retain ecological information and/or to reduce efforts for the identification of organisms and sample processing. One or more species may be selected to form a surrogate following the logic of three unifying macrocriteria, to which any other single selection criterion can be ascribed: *relevance*, *easiness*, and *resemblance*. *Relevance* concerns the importance of a given species, taxon, or group of organisms from an ecological perspective, whether general (e.g., keystone species, habitat formers, bioengineers, conservation targets), context-specific (e.g., tolerant, sensitive, indicator species, or taxa), or study-specific (e.g., species, taxa, or group of organisms most contributing to the observed patterns). *Easiness* relates to the distinctiveness of a given species, taxon, or group of organisms leading to be easily identified from a taxonomic, morphological, or functional point of view, even by nonexpert taxonomists. Finally, *resemblance* concerns shared characteristics among organisms, from common ancestry to functional similarity (e.g., phylogenetically/taxonomically related species, trophic groups) that allow meaningful groupings.

**Figure 2:**
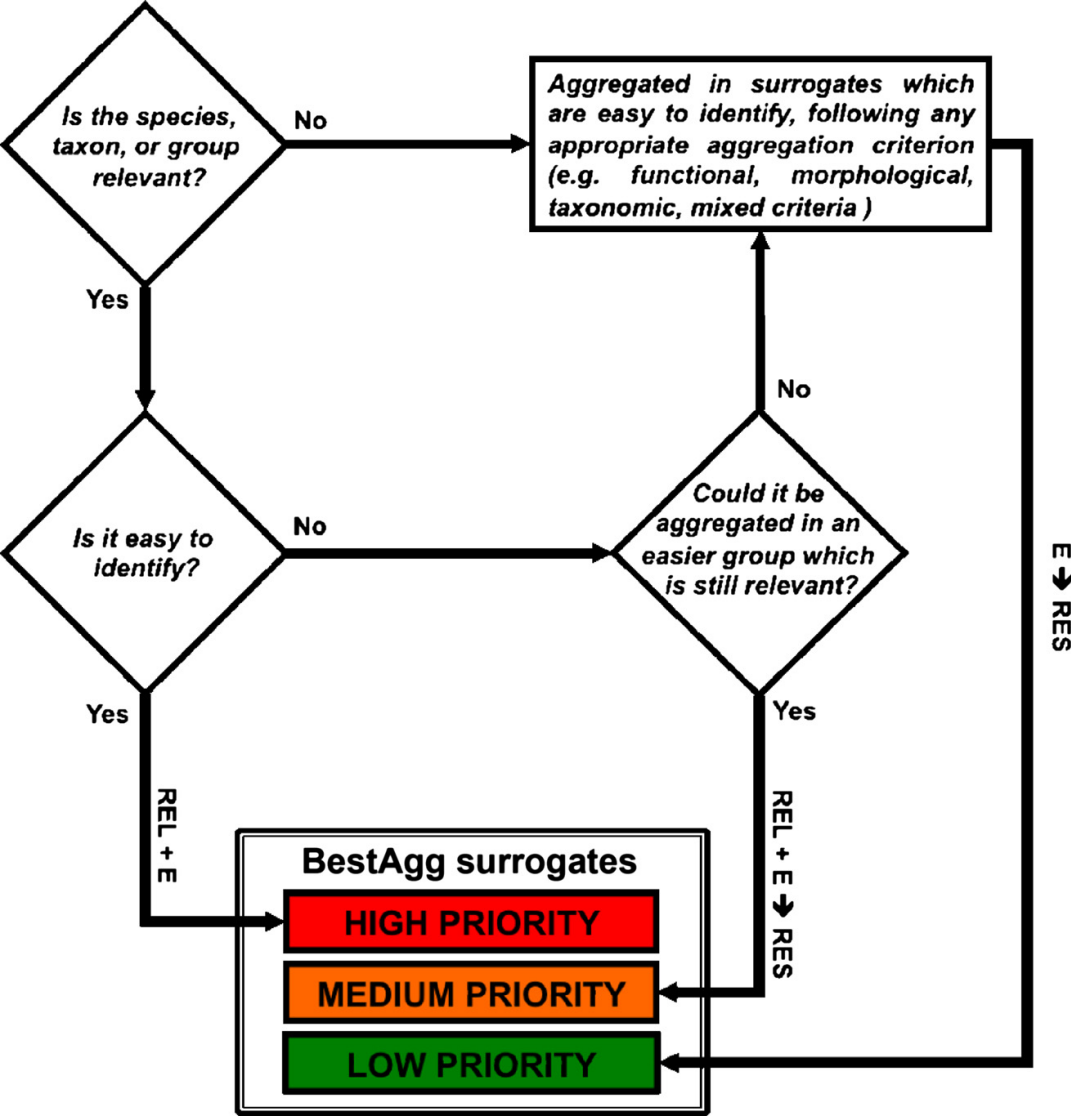
A conceptual framework for surrogate selection in BestAgg. High-priority surrogates are single species (or taxa, groups, etc.) owning the characteristics of *relevance* and *easiness* (REL + E). Medium-priority surrogates are aggregations of species (or taxa, groups) owing the characteristic of *relevance*, but achieving *easiness* through *resemblance* (REL + E → RES). Low-priority surrogates are aggregations of not relevant species (or taxa, groups) achieving *easiness* through *resemblance* (E → RES).

The process of surrogate selection for BestAgg is guided by the interplay among the characteristics of *relevance*, *easiness*, and *resemblance* of each species (Fig. 2). High priority is given to relevant species that are also easy to identify. Such species are directly selected as surrogates (Fig. 2). Relevant species whose identification is difficult are aggregated, if possible, in easy-to-identify but still relevant surrogates; otherwise such species are joined with not relevant ones (Fig. 2). Intermediate priority is given to such surrogates because their *easiness* is achieved through *resemblance* (i.e., through aggregations of species based on their similarities, such as, for instance, morphological, functional, etc.). Finally, not relevant species are grouped to form surrogates following any appropriate aggregation criterion (Fig. 2). Also in this last case *easiness* is achieved through *resemblance* but, as surrogates mostly include not relevant species, low priority is given to them.

Aggregations leading to low-priority surrogates should increase *easiness* taking into account, nevertheless, that at the end of the selection process the number of selected surrogates for BestAgg, namely, *G*_BestAgg_, has to be ≥*G*_min_. It is worth noting here that *G*_min_ represents the effective *minimum* number of surrogates needed. Therefore, if *G*_BestAgg_ > *G*_min_, it should not be reduced necessarily to *G*_min_, especially if *G*_BestAgg_ ≍ *G*_min_, given that further aggregations would have very little effects in reducing efforts of sample processing (e.g., sorting, counting, identification of organisms). This could not be the case if *G*_BestAgg_ >> *G*_min_. In such situations, further aggregations of low-priority surrogates first and, subsequently, if necessary, of medium- and high-priority surrogates may be used to reduce the number of selected surrogates.

Any species, taxon, or group of organisms matching one or more of the above macrocriteria is potentially eligible as surrogate. BestAgg, however, aims to optimizing trade-offs between *relevance*, *easiness*, and *resemblance* in order to maximizing the relevant ecological information while reducing as much as possible the number of surrogates and the difficulty in the identification of organisms. In this view, the selection process of surrogates in BestAgg is structured following two basic principles: (1) prioritizing the choice of surrogates which are at the same time ecologically relevant and easy to identify; (2) aggregating difficult species into surrogates easier to identify (Fig. 2).

### Checking the surrogate set from BestAgg

The effectiveness of BestAgg surrogates can be checked by comparing results from analyses based on the data matrix in which the original species have been aggregated into the BestAgg surrogates with those obtained analyzing the original species-level data matrix. Multivariate community responses using BestAgg surrogates and species should be interchangeable. Moreover, the information retained in the BestAgg aggregated matrix should be within random expectations from the null model. This is because BestAgg assumes that surrogates are random subsets of the original pool of species and, therefore, selected surrogates should work at least as well as random expectations. Indeed, the condition in which the selected surrogates allow retaining significantly greater information on species-level patterns than random expectations is even more desirable, as this would mean that the selection procedure has led to a set of surrogates that are able to represent species-level community patterns even better than what is expected by chance. To check for this, a test based on randomizations can be performed (see Appendix S2 for details). In practice, the original *S* species are randomly aggregated in *G*_BestAgg_ groups, where *G*_BestAgg_ is the number of BestAgg surrogates obtained from surrogate selection (see Fig. 2). Random aggregations are repeated 1000 times. Correlation values (ρ_*i*_) between the original species-level matrix and each *i* randomly aggregated matrix are then calculated to obtain a frequency distribution against which testing ρ_BestAgg_, that is, the correlation between the original species-level matrix and the matrix aggregated using BestAgg surrogates. Also, PERMANOVA is performed on randomly aggregated matrices, and the percentage of tests for the term of interest showing consistent results with those obtained at species level is calculated, representing the *P*-value (i.e., the probability of Type-I error) for *G*_BestAgg_.

When the above conditions are respected, that is, (1) results using BestAgg surrogates are consistent with those obtained using species, (2) ρ_BestAgg_ falls within or above random expectations, and (3) the probability of Type-I error is <0.05, the information provided by BestAgg surrogates may substitute effectively species-level information in subsequent sampling programs or very similar case studies.

### Application of BestAgg to real data

We applied BestAgg to two real case studies including different habitats, organism types, and environmental settings, in order to assess the effectiveness of the approach. The first case study, hereafter OP, focused on impact assessment of offshore gas platforms on soft bottom macrobenthic assemblages. This study included species-level data from 2 eight-leg platforms (namely, P1 and P2) located on mud flats at 90 m depth, in the same geographic area (North Ionian Sea). For each platform, macrobenthic assemblages were sampled at increasing distance from its hard structure (i.e., 300, 1000, and 3000 m), in five sites for each distance. The second case study, hereafter DG, related to assessing patterns of variations in sessile assemblages along a depth gradient in Mediterranean rocky cliffs (South Adriatic Sea). This case study included data on species, but also higher taxa and morphological groups, from a 2-year monitoring program involving four times of sampling (T1, T2, T3, and T4). In each time, sessile assemblages were sampled at three depths (i.e., 5, 15, and 25 m) in four locations, with three sampling sites in each location. Additional information on the data sets is provided in Table S1.

The effectiveness of the BestAgg approach was assessed following two steps: (1) the BestAgg procedure was applied to P1 and T1, which served as pilot assessments of BestAgg surrogates for the OP and the DG case study, respectively; (2) BestAgg surrogates from pilot assessments were used to analyze data from the second platform (i.e., P2) for OP, and from the remaining sampling times (i.e., T2, T3, and T4) for DG, which were used as test studies in order to check whether such surrogates were effective under comparable environmental settings.

Finally, the performance of BestAgg was also compared with results obtained applying a more classical approach for species surrogacy based on the concept of taxonomic sufficiency. Specifically, we assessed the amount of information on species-level community patterns retained in matrices aggregated using taxonomic surrogates versus BestAgg surrogates by applying the randomization test described above. In addition, we estimated savings derived from the application of BestAgg and taxonomic surrogates following the approach proposed by Ferraro and Cole (1995) (see also Thompson et al. 2003). In this approach, savings are estimated by taking the ratio of the number of surrogates to the number of species, assuming that the time spent to identify organisms is proportional to the number of categories (e.g., species, higher taxa, groups) to which they must be assigned.

### Statistical analyses

Two separated null models based on randomly aggregated matrices were constructed employing data matrices from pilot studies, that is, P1 (OP) and T1 (DG), following the procedure described previously. Analyses were done using the R code provided in Appendix S1 (see also Appendix S2). The decreasing ϕ values were obtained by setting *d* to 5% of *S* for P1 (very speciose assemblages, see above) and to 10% of *S* for T1. For the construction of null models, and for subsequent multivariate analyses, PERMANOVA was employed to test for significant effects of the investigated environmental drivers (i.e., distance from platform for OP and depth for DG) on assemblage structure. All analyses were based on Bray–Curtis dissimilarities on untransformed data, using 2000 permutations (see Table S1 for details on designs for analyses).

For each pilot assessment, ϕ_low_ and, consequently, *G*_min_, were identified and a linear regression of ρ values from random aggregations against the corresponding ln(ϕ) was fitted. For each case study, the specific set of BestAgg surrogates based on pilot assessments was determined following the procedure described for surrogate selection (see Fig. 2). Study-specific relevant species (or taxa, groups) most contributing to the observed community patterns were identified through similarity percentage analysis (SIMPER, Clarke 1993); only species whose contribution to dissimilarities was ≥3% were selected.

For each pilot assessment, results from PERMANOVA on species-level data were compared to those obtained from analyses based on data aggregated using BestAgg surrogates to check for their effectiveness. The related test based on random aggregations was performed to check for the amount of information retained in the BestAgg aggregated matrix (see above).

Species-level data from test studies, namely, P2 (OP) and T2, T3, and T4 (DG), were then aggregated using the set of BestAgg surrogates from the corresponding pilot studies. Finally, PERMANOVA was carried out on data aggregated using BestAgg surrogates and results compared with those from analyses at species level.

Data from P1 and T1 were also used for a pilot assessment of the sufficient taxonomic level for analyses, following the logic of taxonomic sufficiency. Therefore, the original species-level matrices were aggregated in taxa of single ranks of the Linnaean taxonomic hierarchy. Then, PERMANOVA on taxonomically aggregated data matrices was carried out to identify the sufficient taxonomic resolution needed to detect significant effects of the investigated environmental driver. Null models and the related test based on randomizations from BestAgg were used to check for the effectiveness of such taxonomic surrogates and the information retained in higher taxon matrices. Finally, test data for each case study (i.e., P2 for OP, and T2, T3, T4, for DG) were analyzed at the respective sufficient taxonomic level, and results were compared with those obtained applying the BestAgg approach.

Analyses were performed using R (R Development Core Team 2010).

## Results

**Figure 3:**
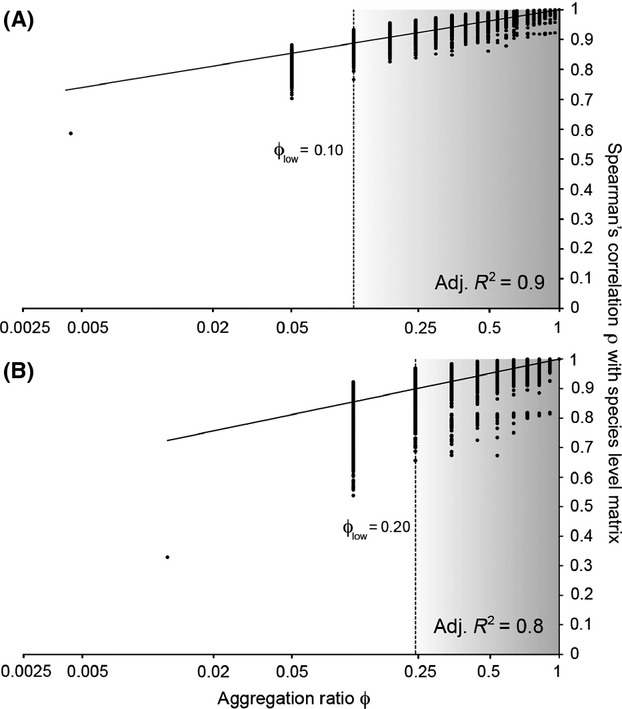
Semilog plot of ρ values between the species-level matrix and each randomly aggregated matrix against the corresponding ϕ values for pilot studies (A) P1 (OP) and (B) T1 (DG). Fading gray zones indicate the range of ϕ values at which analyses were consistent with those at species level. Dotted lines indicate ϕ_low_ (i.e., the lowest practicable aggregation level), sufficient to obtain results consistent with those obtained analyzing species-level data.

For both pilot assessments, linear regressions of ρ against ln(ϕ) were significant (*P* < 0.001), indicating that the information retained in the aggregated matrices strongly depended on the level of aggregation following a semilog model (Fig. 3).

For the OP case study, the pilot assessment showed that the lowest ϕ value allowing 95% of PERMANOVAs on aggregated data to give consistent results with those obtained at species level was ϕ_low_ = 0.10, corresponding to *G*_min_ = 26 (Table 1). For the DG case study, instead, ϕ_low_ = 0.20 and, therefore, *G*_min_ = 16 (Table 1). This means that the original *S* species, that is, 259 for OP and 79 for DG could be aggregated in 26 and 16 surrogates, respectively, while still allowing analyses to perform as well as at species level.

**Table 1:** Percentage of tests from PERMANOVA on random aggregated data consistent with those from species-level analyses, at decreasing levels of aggregation (φ)

OP – Pilot assessment (Platform P1 − *S =* 259)			DG – Pilot assessment (Time 1 − *S =* 79)		
	
Number of surrogates (*G*)	Aggregation ratio (φ)	% Analyses consistent with species level	Number of surrogates (*G*)	Aggregation ratio (φ)	% Analyses consistent with species level
247	0.95	100%	72	0.90	100%
234	0.90	100%	64	0.80	100%
221	0.85	100%	56	0.70	100%
208	0.80	100%	48	0.60	100%
195	0.75	100%	40	0.50	99%
182	0.70	100%	32	0.40	99%
169	0.65	100%	24	0.30	99%
156	0.60	100%	**16**	**0.20**	98%
143	0.55	100%	8	0.10	84%
130	0.50	100%			
117	0.45	100%			
104	0.40	100%			
91	0.35	100%			
78	0.30	100%			
65	0.25	100%			
52	0.20	100%			
39	0.15	100%			
**26**	**0.10**	98%			
13	0.05	69%			

The corresponding number of surrogates (*G*) is also provided. The lowest practicable aggregation (φ)_low_ and the corresponding minimum number of surrogates *G*_min_ are given in bold.

The procedure for selection of BestAgg surrogates from pilot assessments led to define a set of *G*_BestAgg_ = 29 surrogates for OP (see Table S3) and *G*_BestAgg_ = 23 surrogates for DG (see Table S4). General and context-specific ecological relevance of surrogates was defined based on available scientific information (see Tables S3 and S4), whereas study-specific relevance was defined based on species most contributing to the observed patterns (see Table S2 for results of SIMPER analyses). For OP, all 29 surrogates were based on taxonomy, with six species (*Aspidosiphon* sp., *Corbula gibba*, *Golfingia* sp., *Thyasira biplicata*, *Timoclea ovata*, and *Nuculana commutata*), three genera (*Kelliella*, *Diplodonta*, and *Nucula*), four families (Capitellidae, Cirratulidae, Paraonidae, and Spionidae), five orders (Amphipoda, Cumacea, Decapoda, Isopoda, and Tanaidacea), 10 classes (Aplacophora, Asteroidea, Bivalvia, Echinoidea, Gastropoda, Holothuroidea, Polychaeta, Ophiuroidea, Scaphopoda, and Turbellaria), and one phylum (Sipuncula) (Table S3). For DG, the set of 23 BestAgg surrogates was more heterogeneous including 11 taxonomic surrogates from species to class level (*Agelas oroides*, Anthozoa, *Axinella* sp., Bivalvia, Cirripedia, *Cladocora caespitosa*, *Cliona* spp., Hydrozoa, *Peyssonnelia* spp., Tunicates, and *Wrangelia penicillata*), and 12 morphological/functional groups (calcareous tube worms, canopy-forming algae, coarsely branched/unbranched algae, *Crambe*/*Spirastrella*, encrusting Bryozoans, erect Bryozoans, encrusting calcified Rhodophytes, encrusting/massive sponges, green filamentous algae, Madreporarians/Zoanthidea, massive black sponges, and turf-forming Algae) (Table S4).

**Figure 4:**
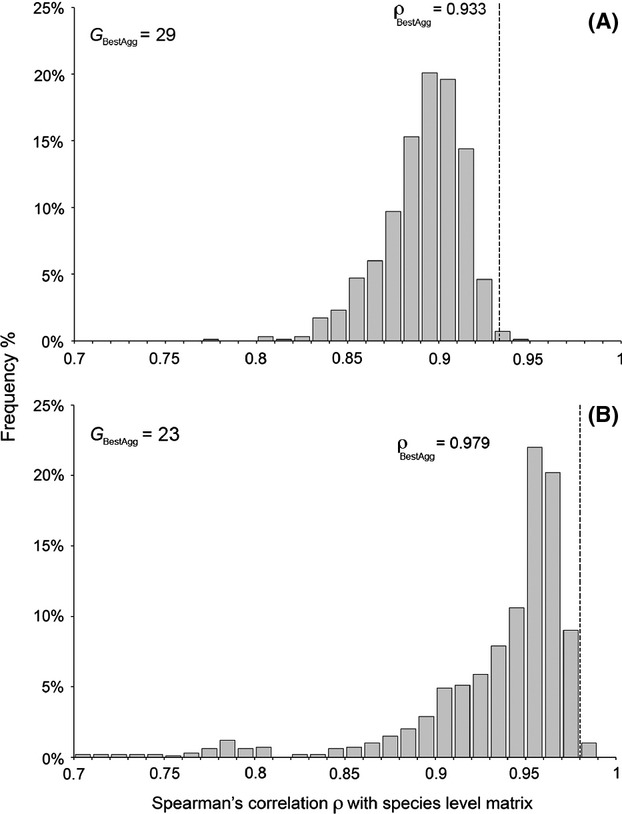
Frequency distribution (*n* = 1000) of ρ values between the species-level matrix and matrices in which species were randomly aggregated in *G*_B__estAgg_ groups (see text and Fig. 2), for pilot studies (A) P1 (OP) and (B) T1 (DG). Dotted lines indicate ρ_BestAgg_, that is, the correlation value between the species-level matrix and the matrix aggregated using BestAgg surrogates, which in both cases fall significantly (*P* < 0.05) above random expectations.

For both pilot assessments, tests based on randomizations showed that the probability of *G*_BestAgg_ to fail in depicting species-level community patterns was *P* = 0.021 for OP and *P* = 0.005 for DG. Correlation ρ_BestAgg_ was in both cases significantly (*P* < 0.05) higher than random expectations (Fig. 4), indicating that data aggregated using BestAgg surrogates retained much more of the original species-level information than what is expected to occur by chance.

Pilot assessments showed that PERMANOVA based on data aggregated using BestAgg surrogates allowed obtaining the same results of species-level analyses (Table 2). For OP, the variance component associated to investigate source of variation (i.e., distance from platform) accounted for 22% of the total variance at species level and 24% when using the BestAgg surrogates. For DG, the variance component associated to investigate source of variation (i.e., depth) accounted for the 26% of the total variance at species level and 27% when using BestAgg surrogates. Analyses on aggregate data were able to detect not only the main effect of the investigated sources of variation but also to depict consistently species-level patterns of difference in community structure along the studied gradients (Table 2). For both case studies, PERMANOVA on test data using the specific BestAgg surrogates derived from pilot assessments showed results consistent with those that would have been obtained if species were analyzed (Table 2).

**Table 2:** Results of PERMANOVA on data aggregated using BestAgg surrogates. Results consistent (including pairwise comparisons) with those obtained at species level (which are also reported) are given in bold

BestAgg pilot assessment				
Case study	Pilot study	Source of variation	Species level	BestAgg
OP	P1	Distance	(*N* ≠ *M* ≠ *F*)***	**(*****N*** **≠** ***M*** **≠** ***F***)***
DG	T1	Depth	(5 ≠ 15 = 25)***	(5 ≠ 15 = 25)***

Application of BestAgg				
Case study	Test study	Source of variation	Species level	BestAgg
OP	P2	Distance	(*N* = *M* ≠ *F*)**	(*N* = *M* ≠ *F*)**
DG	T2	Depth	(5 ≠ 15 = 25)*	(5 ≠ 15 = 25)*
	T3	Depth	(5 ≠ 15 ≠ 25)**	(5 ≠ 15 ≠ 25)**
	T4	Depth	(5 ≠ 15 ≠ 25)**	(5 ≠ 15 ≠ 25)**

P1, Platform 1; P2, Platform 2; *N* = 300 m; *M =* 1000 m; *F* = 3000; T1-2-3-4, Times 1-2-3-4.

*P < 0.05; **P < 0.01; ***P < 0.001.

**Figure 5:**
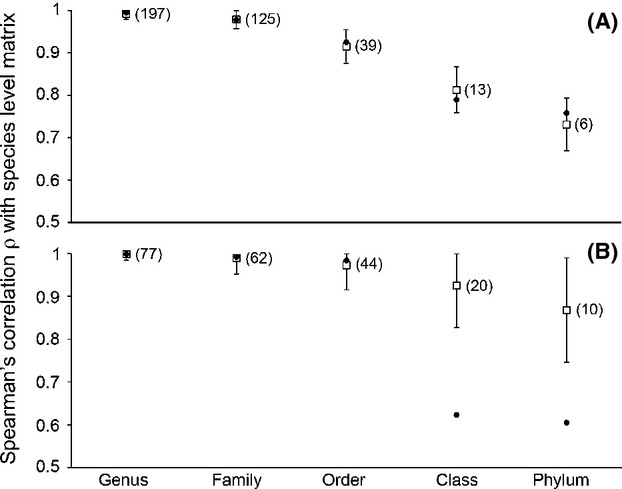
Mean ± 95% confidence interval (*n* = 1000) of ρ values between species and randomly aggregated matrices for (A) P1 pilot study (OP case study) and (B) T1 pilot study (DG case study). Black points are ρ values between species and higher taxon matrices at genus, family, order, class, and phylum level. Numbers in brackets indicate the number of taxa in each taxonomic level.

For both case studies, the information retained in taxonomically aggregated matrices fell within or below the 95% confidence interval from random expectation for all investigated taxonomic levels (i.e., genus, family, order, class, phylum) (Fig. 5), indicating that taxonomic surrogates behaved as, or even worse, than random groups of species. Pilot assessments of taxonomic surrogates based on taxonomic sufficiency indicated order and phylum, respectively, as the sufficient taxonomic levels for analyses in OP and DG (Table 3). In contrast, the sufficient taxonomic level predicted based on the lowest practicable aggregation (ϕ_low_) was that of order for both case studies (Table 3). For OP, PERMANOVA on test data aggregated at order level confirmed this taxonomic resolution as sufficient in providing results consistent with those obtained using species (Table 4). For DG, analyses showed that orders were effective surrogates, whereas the analysis at phylum level, although still detecting main effects, was unsuitable to depict community pattern of difference along the investigated environmental gradient as well as at species level (Table 4).

**Table 3:** *φ* values of each taxonomic level and sufficient *φ* values based on BestAgg (pilot studies)

				Values of *φ* based on taxonomic aggregation								
							
Case study	Pilot study	Source of variation	*S*	Species	Genus	Family	Order	Class	Phylum	Sufficient *φ* from BestAgg	BestAgg prediction of sufficient taxonomic level	Sufficient taxonomic level (classic)
OP	P1	Distance	259	1***	0.76***	0.48***	0.15***	0.06^ns^	0.02^ns^	0.10	Order	Order
DG	T1	Depth	79	1**	0.96**	0.77**	0.55**	0.25*	0.13**	0.20	Order	Phylum

The sufficient taxonomic resolution for analyses based on taxonomic sufficiency (classic approach) and the sufficient taxonomic resolution predicted on the basis of the sufficient *φ* (i.e., *φ*_low_) from BestAgg are provided. The number of species *S* in the original matrix and results of PERMANOVA for each taxonomic level are also reported. P1, Platform 1; T1, Time 1. ns, not significant. *P < 0.05; **P < 0.01; ***P < 0.001.

**Table 4:** Results of PERMANOVA on test data aggregated on the basis of the sufficient taxonomic level determined using taxonomic sufficiency (classic approach) and the lowest practicable aggregation level *φ*_low_ from BestAgg (see Table S5)

Case study	Source of variation	Taxonomic level	Test study		
OP	Distance		P2		
		Species	(*N* = *M* ≠ *F*)**		
		Order	(*N* = *M* ≠ *F*)**		

DG	Depth		*T*2	*T*3	*T*4
		Species	(5 ≠ 15 = 25)*	(5 ≠ 15 ≠ 25)**	(5 ≠ 15 ≠ 25)**
		Order	(5 ≠ 15 = 25)*	**(5 ≠** 15 ≠ 25)**	**(5** **≠** 15 **≠** 25)**
		Phylum	(5 = 15 ≠ 25)*	(5 ≠ 15 = 25)**	(5 ≠ 15 = 25)*

For OP, both approaches indicated the level of order as sufficient. For DG, the classic approach and *φ*_low_ indicated phylum and order, respectively, as the sufficient taxonomic levels. Results consistent (including pairwise comparisons) with those obtained at species level (which are also reported) are given in bold. P2, Platform 2; *N* = 300 m; *M* = 1000 m; *F* = 3000; T2-3-4, Times 2-3-4. *P < 0.05; **P < 0.01; ***P < 0.001.

The application of BestAgg surrogates led to an estimated saving of time during sample processing and organism identification of 90% for OP and 71% for DG, in contrast to savings of 85% and 45% for OP and DG, respectively, when using taxonomic surrogates.

## Discussion

Over the past three decades, the use of higher taxa as surrogates for species has received increasing attention as a pragmatic solution to overcome impediments related to fine taxonomic identifications of organisms in ecological studies (Bevilacqua et al. 2012). However, a number of issues on taxonomic surrogates remained largely unsolved, possibly preventing the consolidation of such a practice in routine monitoring programs despite its undeniable advantages (Dauvin et al. 2007). The use of taxonomic surrogates is problematic when the allocation of species into higher taxa is queried, or when cladistic revisions of the taxonomic hierarchy lead to the insertion/removal of additional (e.g., infraorder, superfamily) or classic ranks. Also, taxa of the same rank may not be equivalent from a phylogenetic point of view among different phyla, making the use of taxonomic surrogates less stringent when considering assemblages embracing more than one phylum (Bertrand et al. 2006; Bevilacqua et al. 2009). More importantly, ecological similarity among species within taxa may be markedly taxon specific (e.g., Losos 2008), hampering the association of a clear ecological meaning to changes in community structure when it is codified through ranks of the Linnaean hierarchy higher than species (Somerfield and Clarke 1995; Terlizzi et al. 2003; Bertrand et al. 2006; Jones 2008).

In spite of these evident intrinsic limits, approaches based on taxonomic relationships have profoundly conditioned the way species surrogacy is conceived so far. The BestAgg framework proposed in this study attempts to rise above this stagnant perspective. BestAgg focuses on the aggregation of variables in multivariate data matrices, looking at the effect of aggregation on congruencies between the information contained in the original versus the corresponding aggregated matrix (Bevilacqua et al. 2012). The approach is based on the simple concept that the higher the level of aggregation (i.e., the ratio of the number of aggregated variables to the number of original variables) the higher the loss of information. As it is the numerical relationship between original and aggregated variables that matters, the nature of variables (which could express the abundance of species, taxa, morphological groups, etc.) and the logic guiding variables’ aggregation are irrelevant. Thus, BestAgg is applicable to any kind of community data from any environmental context and type of organisms (whether involving a single phylum or more different ones), allows mixing any type of surrogates (as the identification of the sufficient number of surrogates goes beyond any potential relationship among species, whether taxonomic, phylogenetic, etc.), and prioritizes the choice of ecologically meaningful groupings (in contrast to taxonomic surrogates, for instance, which are based on taxonomic relatedness regardless of whether higher taxa could actually represent ecologically meaningful units).

It could be argued that such numerical relationships might be biased by sample size, as might happen for ratios between taxonomic categories/subcategories (Gotelli and Colwell 2001). This is not the case for aggregation ratios in BestAgg because (1) the approach does not assume any intrinsic relationship between the original and the aggregated variables (which, actually, are arbitrary categories deriving from random aggregations) and (2) sample size is constant for a given study.

Disentangling species surrogacy from static aggregation schemes, BestAgg, can also take advantage of using different surrogate types (e.g., higher taxa, functional groups, ecological indicators, etc.). Such a prerogative is decisive to open species surrogacy to ecological knowledge (Groc et al. 2010), which can guide the choice of those surrogates more aligned to ecological characteristics of species in order to maximizing ecological information on community patterns notwithstanding the inherent reduction in taxonomic detail. Although this aspect seems to introduce some level of subjectivity in the approach, surrogates selection in BestAgg is far from being arbitrary. The *number* of effective surrogates is determined objectively and the *identity* of surrogates is determined based on objective macrocriteria. Also, evidence from pilot assessments and the solid scientific information on the investigated system substantiate the choice of surrogates, limiting the subjectivity of the experimenter (Dauvin et al. 2007). Moreover, in contrast to other approaches, within the BestAgg framework, the experimenter can set a priori the probability of failing in detecting significant results using the selected surrogates and therefore controlling for uncertainty on their application.

Quite intuitively, the set of surrogates from BestAgg may be strictly context specific because their choice, as for any other approach to species surrogacy, depends on the aim of the study, the particular environmental situation, the organisms involved, and the available ecological knowledge of the system. Pragmatic considerations seem to suggest that levels of aggregation up to 0.4–0.5 (corresponding to a number of surrogates equal to 40–50% of the original number of species) are usually still conducive to effective representations of species-level community patterns (Bevilacqua et al. 2012). However, one-fit-all solutions in species surrogacy could be misleading and the identification of suitable surrogates for a given study needs to be based on representative pilot assessments at species level (Terlizzi et al. 2003; Jones 2008; Siqueira et al. 2012). Therefore, the set of effective surrogates obtained from the application of BestAgg to a pilot study should be applied to subsequent studies in very similar environmental contexts only (e.g., same source of impact in the same habitat, the same natural gradient in areas of the same region, etc.), and given the same experimental design, which, clearly, needs to be appropriately planned to assess the effects of the investigated source of variability in modifying community patterns. We simulated the application of BestAgg to real case studies, in which a first pilot assessment was performed to define the set of effective surrogates that was then used successfully in similar environmental investigations (as in the OP example), or for subsequent monitoring programs (as in the DG example). Results demonstrated the robustness of BestAgg in analyzing community patterns in relation to both natural and human-driven gradients, whether involving individual or colonial species, although further efforts are required to extend this approach to other environmental contexts. As the estimation of cost savings deriving from using surrogates strongly depends on the investigated group(s) of organisms, the number of specimens to be classified, and available taxonomic expertise (Ferraro and Cole 1990), quantifying the advantages provided by BestAgg surrogates in term of costs with respect to classic taxonomic surrogates is a difficult task and estimated cost savings could not have a general validity. However, our results on real case studies showed that BestAgg surrogates might lead gaining up to 25% of time during sample processing with respect to classic taxonomic surrogates. Moreover, such a time saving is likely to be underestimated because, in contrast to taxonomic surrogates, which imply at least a basic taxonomic expertise, the choice of surrogates in BestAgg prioritizes identification easiness and might involve nontaxonomic surrogates (e.g., morphological groups).

Above all, our findings showed that BestAgg represents a valuable alternative method to species surrogacy in environmental impact assessment and ecological monitoring, potentially leading to increased time saving with respect to traditional approaches, such as those involving the use of higher taxa as surrogates for species. In addition, BestAgg recognizes the need for conferring an ecological meaning to surrogates, which is fundamental for the interpretation of ecological patterns. It is increasingly evident that the quest for effective proxies for species has to abandon static approaches, moving toward the integration of taxonomic, phylogenetic, and functional aspects (Devictor et al. 2010). BestAgg may represent a step forward in this direction.

## Supporting Information

**Tables S1.** Dataset information.

**Tables S2.** Results of SIMPER analysis.

**Tables S3.** Selected surrogates for BestAgg in the OP case study.

**Tables S4.** Selected surrogates for BestAgg in the DG case study.

**Appendix S1.** R code for BestAgg analyses.

**Appendix S2.** User guide to the R code.

**Appendix S3.** Example data (as separate .csv file).

**Appendix S4.** Factors for example data (as separate .csv file).

## References

[b1] Anderson MJ (2001). A new method for non-parametric multivariate analysis of variance. Austral Ecol.

[b2] Beattie AJ, Oliver I (1994). Taxonomic minimalism. Trends Ecol. Evol.

[b3] Bertrand Y, Pteijel F, Rouse GW (2006). Taxonomic surrogacy in biodiversity assessments, and the meaning of Linnaean ranks. Syst. Biodivers.

[b4] Bevilacqua S, Fraschetti S, Musco L, Terlizzi A (2009). Taxonomic sufficiency in the detection of natural and human-induced changes in marine assemblages: a comparison of habitats and taxonomic groups. Mar. Pollut. Bull.

[b5] Bevilacqua S, Terlizzi A, Fraschetti S, Claudet J, Boero F (2012). Taxonomic relatedness does not matter for species surrogacy in the assessment of community responses to environmental drivers. J. Appl. Ecol.

[b6] Boero F (2001). Light after dark: the partnership for enhancing expertise in taxonomy. Trends Ecol. Evol.

[b7] Cardoso P, Erwin TL, Borges PAV, New TR (2011). The seven impediments in invertebrate conservation and how to overcome them. Biol. Conserv.

[b8] de Carvalho MR, Bockmann FA, Amorim DS, Brandão CRF, de Vivo M, de Figueiredo JL (2007). Taxonomic Impediment or Impediment to Taxonomy? A commentary on systematics and the cybertaxonomic-automation paradigm. Evol. Biol.

[b9] Clarke KR (1993). Non-parametric multivariate analysis of changes in community structure. Aust. J. Ecol.

[b10] Dauvin J-C, Bellan G, Bellan-Santini D (2007). Benthic indicators: from subjectivity to objectivity – Where is the line?. Mar. Pollut. Bull.

[b11] Defeo O, Lercari D (2004). Testing taxonomic resolution levels for ecological monitoring in sandy beach macrobenthic communities. Aquat. Conserv.

[b12] Dethier MN, Schoch GC (2006). Taxonomic sufficiency in distinguishing natural spatial patterns on an estuarine shoreline. Mar. Ecol. Prog. Ser.

[b13] Devictor V, Mouillot D, Meynard C, Jiguet F, Thuiller W, Mouquet N (2010). Spatial mismatch and congruence between taxonomic, phylogenetic and functional diversity: the need for integrative conservation strategies in a changing world. Ecol. Lett.

[b14] Ferraro SP, Cole FA (1990). Taxonomic level and sample size sufficient for assessing pollution impacts on the Southern California Bight macrobenthos. Mar. Ecol. Prog. Ser.

[b15] Ferraro SP, Cole FA (1995). Taxonomic level sufficient for assessing pollution impacts on the southern Californian Bight macrobenthos – revisited. Environ. Toxicol. Chem.

[b16] Fonseca VG, Carvalho GR, Sung W, Johnson HF, Power DM, Neill SP (2010). Second-generation environmental sequencing unmasks marine metazoan biodiversity. Nat. Commun.

[b17] Fontaine B, Alonso-Zarazaga K, van Achterberg MA, Araujo R, Asche M, Aspöck H (2012). New species in the Old World: Europe as a frontier in biodiversity exploration, a test bed for 21st Century taxonomy. PLoS One.

[b18] Giangrande A, Licciano M, Musco L (2005). Polychaetes as environmental indicators revisited. Mar. Pollut. Bull.

[b19] Gotelli N, Colwell RK (2001). Quantifying biodiversity: procedures and pitfalls in the measurement and comparison of species richness. Ecol. Lett.

[b20] Groc S, Delabie JHC, Longino JT, Orivel J, Majer JD, Vasconcelos HL (2010). A new method based on taxonomic sufficiency to simplify studies on Neotropical ant assemblages. Biol. Conserv.

[b21] Gross M (2012). Barcoding biodiversity. Curr. Biol.

[b22] Heino J, Soininen J (2007). Are higher taxa adequate surrogates for species level assemblage patterns and species richness in stream organisms?. Biol. Conserv.

[b23] Hill D, Arnold R (2012). Building the evidence base for ecological impact assessment and mitigation. J. Appl. Ecol.

[b24] Hooper DU, Adair EC, Cardinale BJ, Byrnes JEK, Hungate BA, Matulich KL (2012). A global synthesis reveals biodiversity loss as a major driver of ecosystem change. Nature.

[b25] Jones FC (2008). Taxonomic sufficiency: the influence of taxonomic resolution on freshwater bioassessments using benthic macroinvertebrates. Environ. Rev.

[b26] Legendre P, Anderson MJ (1999). Distance-based redundancy analysis: testing multispecies responses in multifactorial ecological experiments. Ecol. Monogr.

[b27] Lenat DR, Resh VH (2001). Taxonomy and stream ecology – the benefits of genus- and species-level identifications. J. N. Am. Benthol. Soc.

[b28] Lomolino MV, Lomolino MV, Heaney LR (2004). Conservation biogeography. Frontiers of biogeography: new directions in the geography of nature.

[b29] Losos JB (2008). Phylogenetic niche conservatism, phylogenetic signal and the relationship between phylogenetic relatedness and ecological similarity among species. Ecol. Lett.

[b30] Lovell S, Hamer M, Slotow R, Herbert D (2007). Assessment of congruency across invertebrate taxa and taxonomic levels to identify potential surrogates. Biol. Conserv.

[b31] Mandelik Y, Roll U, Fleischer A (2010). Cost-efficiency of biodiversity indicators for Mediterranean ecosystems and the effects of socio-economic factors. J. Appl. Ecol.

[b32] May RM (1990). Taxonomy as destiny. Nature.

[b33] McArdle BH, Anderson MJ (2001). Fitting multivariate models to community data: a comment on distance-based redundancy analysis. Ecology.

[b34] Mellin C, Delean S, Caley J, Edgar G, Meekan M, Pitcher R (2011). Effectiveness of biological surrogates for predicting patterns of marine biodiversity: a global meta-analysis. PLoS One.

[b35] Neeson T, van Rijn I, Mandelik Y (2013). How taxonomic diversity, community structure and sample size determine the reliability of higher taxon surrogates. Ecol. Appl.

[b36] Pereira HM, Navarro LM, Santos Martins I (2012). Global biodiversity change: the bad, the good, and the unknown. Annu. Rev. Environ. Resour.

[b37] Pik AJ, Oliver I, Beattie AJ (1999). Taxonomic sufficiency in ecological studies of terrestrial invertebrates. Aust. J. Ecol.

[b38] R Development Core Team (2010). R: a language and environment for statistical computing. http://www.R-project.org.

[b39] Scheffer M, Carpenter SR, Lenton TM, Bascompte J, Brock W, Dakos V (2012). Anticipating critical transitions. Science.

[b40] Siqueira T, Bini LM, Roque FO, Cottenie K (2012). A metacommunity framework for enhancing the effectiveness of biological monitoring strategies. PLoS One.

[b41] Snelgrove PVR (2010). Discoveries of the census of marine life: making ocean life count.

[b42] Somerfield PJ, Clarke KR (1995). Taxonomic levels, in marine community studies revisited. Mar. Ecol. Prog. Ser.

[b43] Terlizzi A, Bevilacqua S, Fraschetti S, Boero F (2003). Taxonomic sufficiency and the increasing insufficiency of taxonomic expertise. Mar. Pollut. Bull.

[b44] Terlizzi A, Anderson MJ, Bevilacqua S, Fraschetti S, Włodarska-Kowalczuk M, Ellingsen KE (2009). Beta diversity and taxonomic sufficiency: do higher-level taxa reflect heterogeneity in species composition?. Divers. Distrib.

[b45] Thompson BW, Riddle MJ, Stark JS (2003). Cost-efficient methods for marine pollution monitoring at Casey Station, East Antarctica: the choice of sieve mesh-size and taxonomic resolution. Mar. Pollut. Bull.

[b46] Van Wynsberge S, Andréfouët S, Hamel MA, Kulbicki M (2012). Habitats as surrogates of taxonomic and functional fish assemblages in coral reef ecosystems: a critical analysis of factors driving effectiveness. PLoS One.

[b47] Wägele H, Klussmann-Kolb A, Kuhlmann M, Haszprunar G, Lindberg D, Koch A (2011). The taxonomist - an endangered race. A practical proposal for its survival. Front. Zool.

[b48] Warwick RM (1993). Environmental-impact studies on marine communities – pragmatical considerations. Aust. J. Ecol.

[b49] Wheeler QD (2004). Taxonomic triage and the poverty of phylogeny. Philos. Trans. R. Soc. Lond. B Biol. Sci.

[b50] Wheeler QD, Raven PH, Wilson EO (2004). Taxonomy: impediment or expedient?. Science.

[b51] Whittaker RJ, Araújo MB, Paul J, Ladle RJ, Watson JEM, Willis KJ (2005). Conservation biogeography: assessment and prospect. Divers. Distrib.

[b52] Wilson EO (2003). The encyclopaedia of life. Trends Ecol. Evol.

[b53] Wilson EO (2004). Taxonomy as a fundamental discipline. Philos. Trans. R. Soc. Lond. B Biol. Sci.

[b54] Yu DW, Ji Y, Emerson BC, Wang X, Ye C, Yang C (2012). Biodiversity soup: metabarcoding of arthropods for rapid biodiversity assessment and biomonitoring. Methods Ecol. Evol.

