# Supplementary material for: Best Practicable Aggregation of Species: a step forward for species surrogacy in environmental assessment and monitoring
Source: Ecol Evol. 2013 Sep 11;3(11):3780–93. doi: 10.1002/ece3.715 (PMC3810874; doi:10.1002/ece3.715)
Supplement: Supplementary file 3 [file ece30003-3780-SD3.doc]

**Best Practicable Aggregation of Species (BestAgg): A step forward for species surrogacy in environmental assessment and monitoring**

Stanislao Bevilacqua^1,*^, Joachim Claudet^2,3^, Antonio Terlizzi^1^

^1^Laboratory of Zoology and Marine Biology, Department of Biological and Environmental Sciences and Technologies, University of Salento, 73100 Lecce, Italy; ^2^Laboratoire d’Excellence ‘CORAIL’; ^3^National Center for Scientific Research, USR 3278 CNRS-EPHE CRIOBE, University of Perpignan, 66860 Perpignan Cedex, France

*Corresponding author: stanislao.bevilacqua@unisalento.it

**Supplementary Information**

Additional material complementing the article “*Best Practicable Aggregation of Species (BestAgg): A step forward for species surrogacy in environmental assessment and monitoring*” by Bevilacqua *et al.* is provided in this section. Supplementary Information consists of:

**4 TABLES**

**Tables S1.** Dataset information;

**Tables S2.** Results of SIMPER analysis;

**Tables S3.** Selected surrogates for BestAgg in the OP case study;

**Tables S4.** Selected surrogates for BestAgg in the DG case study;

**4 APPENDICES**

**Appendix S1.** R code for BestAgg analyses;

**Appendix S2.** User guide to the R code;

**Appendix S3.** Example data (as separate .csv file);

**Appendix S4.** Factors for example data (as separate .csv file);

**Table S1.** Dataset information for the Offshore Platform (OP) and the Depth Gradient (DG) case studies.

|  | OP |  | DG |
| --- | --- | --- | --- |
| Location | North Ionian (Mediterranean Sea) |  | South Adriatic (Mediterranean Sea) |
| Habitat | Continental shelf mud flats |  | Subtidal rocky cliffs |
| Type of organisms | Soft bottom macrobenthos |  | Hard bottom sessile macrobenthos |
| Environmental context | Offshore gas fields |  | Natural environmental gradient |
| Source of variation | Distance from offshore platform |  | Depth |
| Datasets | Platform 1 (P1), Platform 2 (P2) |  | Time 1 (T1), Time 2 (T2), Time 3 (T3), Time 4 (T4) |
| Total number of samples | 90 |  | 480 |
| Number of species, taxa, or groups (*S*) | *S*_P1_ = 259, S_P2_ = 113 |  | *S*_T1_ = 79, S_T2_ = 62, *S*_T3_ = 76,  *S*_T4_ = 75 |
| Sampling method | 0.1 m^2^ Van Veen grab |  | 16 × 25 cm photographic frame |
| Taxonomic resolution | Species |  | Species (85%), higher taxa o morphological groups (15%) |
| Experimental design | Distance (fixed, 3 levels), Site (random, 5 levels, nested in Distance) |  | Depth (fixed, 3 levels), Location (random, 4 levels, crossed), Site (random, 3 levels, nested in Location) |
| Reference | Terlizzi, A., Bevilacqua, S., Scuderi, D., Fiorentino, D., Guarnieri, G., Giangrande, A., Licciano, M., Felline, S. & Fraschetti, S. (2008). Effects of offshore platforms on softbottom macro-benthic assemblages: A case study in a Mediterranean gas field. *Marine Pollution Bulletin*, 56,1303–1309. |  | Terlizzi, A., Anderson, M.J., Fraschetti, S. & Benedetti-Cecchi, L. (2007) Scales of spatial variation in Mediterranean subtidal sessile assemblages at different depths. *Marine Ecology Progress Series*, 332, 25–39. |

**Table S2.** Results of SIMPER analysis reporting the % contribution of species (or taxa, groups) to assemblage dissimilarities between different distances (i.e. 300 m, 1000 m, 3000 m) from platform P1 (OP case study) and between different depths (i.e. 5 m, 15 m, 25 m) in sampling time T1 (DG case study). Only species with contributions higher than 3% in at least one pair-wise comparison are reported. Numbers in brackets are average dissimilarities between assemblages.

| P1 (OP) | 300m *vs* 1000m |  | 300m *vs* 3000m |  | 1000m *vs* 3000m |
| --- | --- | --- | --- | --- | --- |
|  | (68.25) |  | (78.28) |  | (79.12) |
| Species |  |  |  |  |  |
| *Aricidea catherinae* | 3.95 |  | - |  | - |
| *Corbula gibba* | 3.29 |  | - |  | - |
| *Diplodonta trigona* | 11.23 |  | 11.05 |  | 4.94 |
| *Golfingia* sp. | 3.09 |  | - |  | - |
| *Kelliella abyssicola* | 6.26 |  | 6.81 |  | 7.00 |
| *Levinsenia gracilis* | 4.95 |  | 3.12 |  | - |
| *Saccella (Jupiteria) commutata* | - |  | 4.5 |  | 5.42 |
| *Parvicardium minimum* | - |  | 3.53 |  | 4.32 |
| *Prionospio cirrifera* | - |  | - |  | - |
| *Thyasira biplicata* | 4.12 |  | 4.17 |  | - |
| *Timoclea ovata* | 7.46 |  | 19.01 |  | 22.18 |
|  |  |  |  |  |  |
|  |  |  |  |  |  |
| T1 (DG) | 5m *vs* 15m |  | 5m *vs* 25m |  | 15m *vs* 25m |
|  | (75.31) |  | (78.83) |  | (53.53) |
| Species |  |  |  |  |  |
| *Agelas oroides* | - |  | 3.12 |  | 4.04 |
| *Cliona* spp. | - |  | - |  | 3.19 |
| *Crambe crambe* | 3.25 |  | 3.11 |  | - |
| *Dictyota dichotoma* | 5.15 |  | 4.81 |  | 9.44 |
| Encrusting Calcified Rhodophyta | 9.63 |  | 9.4 |  | 7.62 |
| Encrusting Bryozoans | 4.01 |  | - |  | 4.87 |
| Green Filamentous Algae | 7.75 |  | 6.44 |  | 9.56 |
| Massive Dark Sponges | 3.65 |  | 3.51 |  | - |
| *Peyssonnelia* sp. | 32.74 |  | 33.03 |  | 29.36 |
| Thin Tubular Sheet-like Algae | - |  | - |  | 3.78 |
| *Wrangelia penicilata* | 3.5 |  | 3.46 |  | - |

**Table S3.** Selected surrogates for BestAgg in the OP case study (see Method section, see also Appendix S5). *Relevance* is reported according to evidence from literature and SIMPER analysis (see Method section, see also Table S2). For *Easiness*: y = easy identification, n = difficult identification. In the *Resemblance* column are reported aggregation criteria if applied (NA = not applied). Priority has been assigned following the procedure described in Appendix S5. Numbers in brackets are the number of species included in taxa and surrogates.

| **Phylum** | **Species, taxon, group** | ***Relevance*** | ***Easyness*** | ***Resemblance*** | **Priority** | **BestAgg surrogate** |
| --- | --- | --- | --- | --- | --- | --- |
| Mollusca (157) | *Corbula (Varicorbula) gibba* | Tolerant/indicator^1^ SIMPER | y | NA | High | *Corbula (Varicorbula) gibba* (1) |
|  | *Thyasira biplicata* | Tolerant/indicator^1^ SIMPER | y | NA | High | *Thyasira biplicata* (1) |
|  | *Timoclea ovata* | Tolerant/indicator^1^ SIMPER | y | NA | High | *Timoclea ovata* (1) |
|  | *Nucula sulcata* | Sensitive^1^ SIMPER | n | Easier at genus level: aggregated with other 2 congenerics in *Nucula* | Medium | *Nucula* (3) |
|  | *Saccella (Jupiteria) commutata* | SIMPER | y | NA | High | *Saccella (Jupiteria) commutata* (1) |
|  | *Diplodonta trigona* | SIMPER | n | Easier at genus level: aggregated in *Diplodonta* | Medium | *Diplodonta* (1) |
| Mollusca (157) | *Kelliella abyssicola* | SIMPER | n | Easier at genus level: aggregated in *Kelliella* | Medium | *Kelliella* (1) |
|  | *Parvicardium minimum* | SIMPER | n | Very difficult: aggregated in Bivalvia (see Other Mollusca) | Low | Bivalvia (52) |
|  | Other Mollusca (148) | Not relevant | n | Very easy at class level: aggregated in Aplacophora, Gastropoda, Bivalvia, Scaphoda | Low | Aplacophora (2) |
|  |  |  |  |  | Low | Gastropoda (90) |
|  |  |  |  |  | Low | Bivalvia (52) |
|  |  |  |  |  | Low | Scaphopoda (4) |
| Annelida (45) | *Aricidea catherinae* | SIMPER | n | Easier at family level: aggregated in Paraonidae | Medium | Paraonidae (2) |
|  | *Levinsenia gracilis* |  |  |  |  |  |
|  | *Prionospio malmgreni* | Tolerant/indicator^1^ | n | Easier at family level: aggregated in Spionidae (tolerant/indicator)^1^ | Medium | Spionidae (3) |
|  | *Prionospio cirrifera* | Tolerant/indicator^1^ SIMPER | n | Easier at family level: aggregated in Spionidae (tolerant/indicator)^1^ | Medium | Spionidae (3) |
|  | *Prionospio* sp. | Tolerant/indicator^1^ |  |  |  |  |
| Annelida (45) | *Chaetozone* sp. | Tolerant/indicator^1^ | n | Easier at family level: aggregated in Cirratulidae (tolerant/indicator)^1^ | Medium | Cirratulidae (2) |
|  | *Monticellina dorsobranchialis* |  |  |  |  |  |
|  | *Pseudoleiocapitella fauveli* | Tolerant/indicator^1^ | n | Easier at family level: aggregated in Capitellidae (tolerant/indicator)^1^ | Medium | Capitellidae (2) |
|  | *Notomastus latericeus* |  |  |  |  |  |
|  | Other Annelida (36) | Not relevant | n | Very difficult: aggregated in Polychaeta (except for Paraonidae, Cirratulidae, Spionidae, and Capitellidae, see above) | Low | Polychaeta (36) |
| Sipuncula (3) | *Golfingia* sp. | Sensitive^1^ SIMPER | y | NA | High | *Golfingia* sp. (1) |
|  | *Aspidosiphon* sp. | Sensitive^1^ | y | NA | High | *Aspidosiphon* sp. (1) |
| Sipuncula (3) | Other Sipuncula (1) | Not relevant | n | Difficult: aggregated in Sipuncula | Low | Sipuncula (1) |
| Arthropoda (44) | Crustacea | Not relevant | n | Very easy at order level: aggregated in Tanaidacea, Cumacea, Isopoda, Amphipoda, Decapoda | Low | Tanaidacea (2) |
|  |  |  |  |  | Low | Cumacea (7) |
|  |  |  |  |  | Low | Isopoda (4) |
|  |  |  |  |  | Low | Amphipoda (26) |
|  |  |  |  |  | Low | Decapoda (5) |
| Platyhelminthes (1) | Turbellaria (1) | Not relevant | n | Difficult: aggregated in Turbellaria | Low | Turbellaria (1) |
| Echinodermata (9) | Echinodermata (9) | Not relevant | n | Very easy at class level: aggregated in Echinoidea, Asteroidea, Ophiuroidea, Holothuroidea | Low | Echinoidea (1) |
|  |  |  |  |  | Low | Asteroidea (1) |
| Echinodermata (9) | Echinodermata (9) | Not relevant | n | Very easy at class level: aggregated in Echinoidea, Asteroidea, Ophiuroidea, Holothuroidea | Low | Ophiuroidea (2) |
|  |  |  |  |  | Low | Holothuroidea (5) |

^1^ Literature references for sensitive, tolerant, or indicator species:

Borja, A., Franco, J. & Pérez, V. (2000) A marine biotic index to establish the ecological quality of soft-bottom benthos within European estuarine and coastal environments. *Marine Pollution Bulletin*, **40**, 1100–1114.

Giangrande, A., Licciano, M. & Musco, L. (2005) Polychaetes as environmental indicators revisited. *Marine Pollution Bulletin*, **50**, 1153–1162.

Gray, J.S. & Pearson, T.H. (1982) Objective selection of sensitive species indicative of pollution-induced change in benthic communities. I. Comparative methodology. *Marine Ecology Progress Series*, **9**, 111–119.

Pearson, T. H. & Rosenberg, R. (1978) Macrobenthic succession in relation to organic enrichment and pollution of the marine environment. *Oceanographic and Marine Biology Annual Review*, **16**, 229–311.

Simboura, N. & Zenetos, A. (2002) Benthic indicators to use in Ecological Quality classification of Mediterranean soft bottom marine ecosystems, including a new Biotic Index. *Mediterranean Marine Science*, **3/2**, 77–111.

**Table S4.** Selected surrogates for BestAgg in the DG case study (see Method section, see also Appendix S5). *Relevance* is reported according to evidence from literature and SIMPER analysis (see Method section, see also Table S2). For *Easiness*: y = easy identification, n = difficult identification. In the *Resemblance* column are reported aggregation criteria if applied (NA = not applied). Priority has been assigned following the procedure described in Appendix S5. Numbers in brackets are the number of species (or taxa, groups) included in taxa and surrogates.

| **Phylum** | **Species, taxon, group** | **Relevance** | **Easiness** | **Resemblance** | **Priority** | **BestAgg surrogate** |
| --- | --- | --- | --- | --- | --- | --- |
| Porifera (17) | *Agelas oroides* | SIMPER | y | NA | High | *Agelas oroides* (1) |
|  | *Cliona* spp. | SIMPER | y | NA | High | *Cliona* spp. (1) |
|  | *Crambe*/*Spirastrella* | SIMPER | y | NA | High | Crambe/Spirastrella (1) |
|  | Massive Black Sponges | SIMPER | y | NA | High | Massive Black Sponges (1) |
|  | *Axinella* sp. | Typical of higher depths, protected^1^ | y | NA | High | *Axinella* sp. (1) |
|  | Other Porifera (12) | Not relevant | n | Difficult at species level, high plasticity of colony forms: aggregated in the morphological group Encrusting/Massive Sponges. | Low | Encrusting/Massive Sponges (12) |
| Cnidaria (11) | *Cladocora caespitosa* | Ecological role as biocostructor, madreporarian | y | NA | High | *Cladocora caespitosa* (1) |
|  | *Leptopsammia pruvoti* | Typical of higher depths, madreporarian | n | Aggregated in a taxonomic group with functional/ecological relevance: Madreporarians/Zoanthidea | Medium | Madreporarians/Zoanthidea (5) |
|  | *Parazoanthus axinellae* | Typical of higher depths |  |  |  |  |
|  | *Polycyathus muellerae* | Typical of higher depths, madreporarian |  |  |  |  |
|  | *Balanophyllia europaea* | Madreporarian |  |  |  |  |
|  | *Caryophyllia smithii* | Typical of higher depths, madreporarian |  |  |  |  |
|  | Hydrozoa | Not relevant | y | NA | Low | Hydrozoa (1) |
|  | Other Cnidaria (4) | Not relevant | n | All anthozoans. Aggregated at Class level: Anthozoa | Low | Anthozoa (4) |
| Annelida (1) | Serpulids | Not relevant | n | Sometimes problem in separating from vermetid: aggregated in the morphological group Calcareous tube worms | Low | Calcareous tube worms (2) |
| Mollusca (4) | Vermetidae | Not relevant | n | Sometimes problem in separating from vermetid: aggregated in the morphological group Calcareous tube worms | Low | Calcareous tube worms (2) |
|  | Other Mollusca (3) | Not relevant | n | All bivalves. Aggregated at Class level: Bivalvia | Low | Bivalvia (3) |
| Arthropoda (1) | *Balanus perforatus* | Not relevant | n | Difficult to species level, all sessile crustaceans. Aggregated in the Infraclass Cirripedia | Low | Cirripedia (1) |
| Bryozoa (7) | Encrusting Bryozoans | Not relevant | y | NA | Low | Encrusting Bryozoans (1) |
|  | Other Bryozoans | Typical of higher depths | n | All erect forms typical of higher depths: aggregated in the morphological group Erect Bryozoans | Medium | Erect Bryozoans (6) |
| Chordata (7) | Tunicata | Not relevant | n | Sometime difficult at species level, solitary and colonial forms: aggregated in the taxonomic group Tunicates | Low | Tunicates (7) |
| Algae (31) | Encrusting Calcified Rhodophytes (ECR) | SIMPER | y | NA | High | ECR (1) |
|  | *Peyssonnelia* spp. | SIMPER | y | NA | High | *Peyssonnelia* spp. (1) |
|  | Green Filamentous Algae (GFA) | SIMPER | y | NA | High | GFA (1) |
| Algae (31) | *Wrangelia penicillata* | SIMPER | y | NA | High | *Wrangelia penicillata* (1) |
|  | *Cystoseira* spp. | Ecological role as habitat-forming species, protected^1^ | n | Aggregated in the morpho-functional group Canopy-forming algae | Medium | Canopy-forming algae (6) |
|  | *Dictyota* spp. | SIMPER, ecological role as canopy-forming algae |  |  |  |  |
|  | *Dictyopteris polypodioides* | Ecological role as canopy-forming algae |  |  |  |  |
|  | *Sargassum* sp. | Ecological role as canopy-forming algae |  |  |  |  |
|  | *Laurencia* spp. | Ecological role as canopy-forming algae |  |  |  |  |
|  | *Sphaerococcus coronopifolius* | Ecological role as canopy-forming algae |  |  |  |  |
|  | Other Algae (21) | Not relevant | n | In most of cases difficult at species level, aggregated in two morpho-functional groups: Turf-forming algae and Coarsely branched/unbranched algae | Low | Turf-forming algae (12) |
|  |  |  |  |  | Low | Coarsely branched/unbranched algae (9) |

^1^Bern Convention. Convention on the Conservation of European Wildlife and Natural Heritage. Bern, Switzerland. Council of Europe, 1979.

SPA/BD Protocol. Protocol concerning specially protected areas and biologic al diversity in the Mediterranean. Barcelona Convention, 1992.

**Appendix S1. R code for BestAgg analyses.**

################################################################################

# 1. Create the database format from Species x Sample matrix #

################################################################################

#Reading the file to transform

tab=read.table("Data.csv",sep=";",header=T,strip.white=T,quote="",na.strings="NA")

#Creating the list of species

Sample=names(tab)[7:ncol(tab)]

#Creating empty formatted table

tabres=as.data.frame(matrix(data=NA,nrow=0,ncol=8))

names(tabres)=c("Species", "Genus", "Family", "Order", "Class", "Phylum", "Sample","Ab")

#Creating empty sub-table of missing variables

tabNA=data.frame(matrix(data=NA,nrow=length(Sample),ncol=1))

names(tabNA)=c("Sample")

tabNA$Sample=Sample

#Fillling the empty table with values from the original table

for (i in 1:nrow(tab))

{

#Taking each replicate one by one

tabTemp=tab[i,1:6]

#Replicating each replicate by the number of species

tabTemp=as.data.frame (lapply(tabTemp,rep,nrow(tabNA)))

#Taking abundances of the species

Ab=t(tab[i,7:ncol(tab)])

dimnames(Ab)=NULL

#Creating the table of each replicate

tabTemp=cbind(tabTemp,tabNA,Ab)

#Putting the sub-table of each replicate in the final table

tabres=rbind(tabres,tabTemp)

}

#Adding factors

tabfac=read.table("Factors.csv",sep=";",header=T,strip.white=T,quote="",na.strings="NA")

rownames(tabfac)=NULL

####

FactorA=rep(tabfac[,1],nrow(tab))

FactorB=rep(tabfac[,2],nrow(tab))

FactorC=rep(tabfac[,3],nrow(tab))

tabfac=cbind(FactorA,FactorB,FactorC)

####

tabres=cbind(tabres,tabfac)

#Exporting the table

write.table(tabres,"Data.o.csv",sep=";",row.names=F,col.names=T,quote=F)

rm(list=ls(all=TRUE))

################################################################################

# 2. Calculating simulated correlation and PERMANOVA results for #

# each group of random surrogates #

################################################################################

#

#########################################

# Step 2.1 Build sub-groups table #

#########################################

#Determine number of sub-groups for each dataset

Data.groups=c(247,234,221,208,195,182,169,156,143,130,117,104,91,78,65,52,39,26,13)

#

#########################################

#Step 2.2 Loading required functions #

#########################################

#Source used function

library(reshape)

library(vegan)

myAdonis=function (formula, data = NULL, permutations = 999, method = "bray",

strata = NULL, contr.unordered = "contr.sum", contr.ordered = "contr.poly",

mySS.Res = 1, mydf.Res = 1, myIndex=1, ...)

{

TOL <- 1e-07

Terms <- terms(formula, data = data)

lhs <- formula[[2]]

lhs <- eval(lhs, data, parent.frame())

formula[[2]] <- NULL

rhs.frame <- model.frame(formula, data, drop.unused.levels = TRUE)

op.c <- options()$contrasts

options(contrasts = c(contr.unordered, contr.ordered))

rhs <- model.matrix(formula, rhs.frame)

options(contrasts = op.c)

grps <- attr(rhs, "assign")

qrhs <- qr(rhs)

rhs <- rhs[, qrhs$pivot, drop = FALSE]

rhs <- rhs[, 1:qrhs$rank, drop = FALSE]

grps <- grps[qrhs$pivot][1:qrhs$rank]

u.grps <- unique(grps)

nterms <- length(u.grps) - 1

H.s <- lapply(2:length(u.grps), function(j) {

Xj <- rhs[, grps %in% u.grps[1:j]]

qrX <- qr(Xj, tol = TOL)

Q <- qr.Q(qrX)

tcrossprod(Q[, 1:qrX$rank])

})

if (inherits(lhs, "dist"))

dmat <- as.matrix(lhs^2)

else {

dist.lhs <- as.matrix(vegdist(lhs, method = method, ...))

dmat <- dist.lhs^2

}

n <- nrow(dmat)

I <- diag(n)

ones <- matrix(1, nrow = n)

A <- -(dmat)/2

G <- -0.5 * dmat %*% (I - ones %*% t(ones)/n)

SS.Exp.comb <- sapply(H.s, function(hat) sum(G * t(hat)))

SS.Exp.each <- c(SS.Exp.comb - c(0, SS.Exp.comb[-nterms]))

H.snterm <- H.s[[nterms]]

if (length(H.s) > 1)

for (i in length(H.s):2) H.s[[i]] <- H.s[[i]] - H.s[[i -

1]]

SS.Res <- sum(G * t(I - H.snterm))

df.Exp <- sapply(u.grps[-1], function(i) sum(grps == i))

df.Res <- n - qrhs$rank

if (inherits(lhs, "dist")) {

beta.sites <- qr.coef(qrhs, as.matrix(lhs))

beta.spp <- NULL

}

else {

beta.sites <- qr.coef(qrhs, dist.lhs)

beta.spp <- qr.coef(qrhs, as.matrix(lhs))

}

colnames(beta.spp) <- colnames(lhs)

colnames(beta.sites) <- rownames(lhs)

F.Mod <- (SS.Exp.each/df.Exp)/(mySS.Res/mydf.Res)

f.test <- function(H, myH, G, I, df.Exp, mydf.Res, H.snterm) {

(sum(G * t(H))/df.Exp)/(sum(G * t(myH))/mydf.Res)

}

# SS.perms <- function(H, G, I) {

# c(SS.Exp.p = sum(G * t(H)), S.Res.p = sum(G * t(I - H)))

# }

if (missing(strata))

strata <- NULL

p <- sapply(1:permutations, function(x) permuted.index(n,

strata = strata))

f.perms <- sapply(1:nterms, function(i) {

sapply(1:permutations, function(j) {

f.test(H.s[[i]],myH=H.s[[myIndex]] , G[p[, j], p[, j]], I, df.Exp[i],

mydf.Res, H.snterm)

})

})

SumsOfSqs = c(SS.Exp.each, mySS.Res, sum(SS.Exp.each) + mySS.Res)

tab <- data.frame(Df = c(df.Exp, mydf.Res, n - 1), SumsOfSqs = SumsOfSqs,

MeanSqs = c(SS.Exp.each/df.Exp, mySS.Res/mydf.Res, NA), F.Model = c(F.Mod,

NA, NA), R2 = SumsOfSqs/SumsOfSqs[length(SumsOfSqs)],

P = c((rowSums(t(f.perms) > F.Mod) + 1)/(permutations +

1), NA, NA))

rownames(tab) <- c(attr(attr(rhs.frame, "terms"), "term.labels")[u.grps],

"Residuals", "Total")

colnames(tab)[ncol(tab)] <- "Pr(>F)"

out <- list(aov.tab = tab, call = match.call(), coefficients = beta.spp,

coef.sites = beta.sites, f.perms = f.perms, model.matrix = rhs,

terms = Terms)

class(out) <- "adonis"

out

}

###############################################################################

#Step 2.3a Calculating correlations and PERMANOVA 2 NESTED FACTORS #

###############################################################################

#

#Reading original table

tabOrSpe=read.table("Data.o.csv",sep=";",header=T,strip.white=T,na.strings="NA",quote="")

#Obtaining the original triangular matrix at the species level

tabSpe=cast(tabOrSpe[,c(1:8)],Species+Genus+Family+Order+Class+Phylum~Sample)

tabSpeTri=vegdist(t(tabSpe[,c(7:ncol(tabSpe))]),method="bray")

#Creating empty table with correlations and PERMANOVA results

Data.res=as.data.frame(matrix(data=NA,nrow=1000,ncol=length(Data.groups)*2))

names(Data.res)=c(paste(as.character(Data.groups),"cor",sep="."),paste(as.character(Data.groups),"aov",sep="."))

#Loop on each aggregation levels

for (i in 1:length(Data.groups))

{

#Number of aggregation levels

AggNb=Data.groups[i]

AggIndex=seq(from=1,to=AggNb,by=1)

#Random assignments of simulated assignements on Species

vecAggCorSim=numeric(0)

vecAggPermSim=numeric(0)

for (j in 1:1000)

{

AggSim=sample(AggIndex,length(AggIndex),replace=F)

AggSimTemp=sample(AggIndex,nrow(tabSpe)-length(AggIndex),replace=T)

AggSim=c(AggSim,AggSimTemp)

rm(AggSimTemp)

AggSim=sample(AggSim,length(AggSim),replace=F)

tabSpeSim=cbind(AggSim,tabSpe)

tabAggSim=aggregate(tabSpeSim[,c(8:ncol(tabSpeSim))],by=list(tabSpeSim$AggSim),sum)

#Correlations between simulated assigments and original species

tabAggSimTriang=vegdist(t(tabAggSim[,c(2:ncol(tabAggSim))]),method="bray")

tabAggCorSim=cor(tabSpeTri,tabAggSimTriang,method="spearman")

vecAggCorSim=c(vecAggCorSim,tabAggCorSim)

#Permanova for each simulated assignement

#Creating the right format of the aggregated simulated matrix for permanova

tabAggSimPerm=t(tabAggSim[,c(2:ncol(tabAggSim))])

FactorA=tabOrSpe[match(rownames(tabAggSimPerm),tabOrSpe$Sample),9]

FactorA=as.factor(FactorA)

FactorB=tabOrSpe[match(rownames(tabAggSimPerm),tabOrSpe$Sample),10]

FactorB=as.factor(FactorB)

#First permanova to obtain denominator SS and df

FirstPermanova=adonis(tabAggSimPerm~FactorA+FactorB%in%FactorA,permutations=3,method="bray",strata=NULL)

mydf=FirstPermanova$aov.tab[2,1]

mySS=FirstPermanova$aov.tab[2,2]

#Second permanova with appropriate denominator

SecondPermanova=myAdonis(tabAggSimPerm~FactorA+FactorB%in%FactorA,permutations=2000,method="bray",strata=FactorB%in%FactorA,mySS.Res=mySS,mydf.Res=mydf,myIndex=2)

PermanovaRes=SecondPermanova$aov.tab[1,6]

vecAggPermSim=c(vecAggPermSim,PermanovaRes)

print(c(i,j))

}

#Put correlations in corresponding table assignement group

Data.res[,i]=vecAggCorSim

Data.res[,length(Data.groups)+i]=vecAggPermSim

}

################################################################################

#Step 2.3b Calculating correlations and PERMANOVA 3 NESTED FACTORS #

################################################################################

#

#Reading original table

tabOrSpe=read.table("Data.o.csv",sep=";",header=T,strip.white=T,na.strings="NA",quote="")

#Obtaining the original triangular matrix at the species level

tabSpe=cast(tabOrSpe[,c(1:8)],Species+Genus+Family+Order+Class+Phylum~Sample)

tabSpeTri=vegdist(t(tabSpe[,c(7:ncol(tabSpe))]),method="bray")

#Creating empty table with correlations and PERMANOVA results

Data.res=as.data.frame(matrix(data=NA,nrow=1000,ncol=length(Data.groups)*2))

names(Data.res)=c(paste(as.character(Data.groups),"cor",sep="."),paste(as.character(Data.groups),"aov",sep="."))

#Loop on each aggregation levels

for (i in 1:length(Data.groups))

{

#Number of aggregation levels

AggNb=Data.groups[i]

AggIndex=seq(from=1,to=AggNb,by=1)

#Random assignments of simulated assignements on Species

vecAggCorSim=numeric(0)

vecAggPermSim=numeric(0)

for (j in 1:1000)

{

AggSim=sample(AggIndex,length(AggIndex),replace=F)

AggSimTemp=sample(AggIndex,nrow(tabSpe)-length(AggIndex),replace=T)

AggSim=c(AggSim,AggSimTemp)

rm(AggSimTemp)

AggSim=sample(AggSim,length(AggSim),replace=F)

tabSpeSim=cbind(AggSim,tabSpe)

tabAggSim=aggregate(tabSpeSim[,c(8:ncol(tabSpeSim))],by=list(tabSpeSim$AggSim),sum)

#Correlations between simulated assigments and original species

tabAggSimTriang=vegdist(t(tabAggSim[,c(2:ncol(tabAggSim))]),method="bray")

tabAggCorSim=cor(tabSpeTri,tabAggSimTriang,method="spearman")

vecAggCorSim=c(vecAggCorSim,tabAggCorSim)

#Permanova for each simulated assignement

#Creating the right format of the aggregated simulated matrix for permanova

tabAggSimPerm=t(tabAggSim[,c(2:ncol(tabAggSim))])

FactorA=tabOrSpe[match(rownames(tabAggSimPerm),tabOrSpe$Sample),9]

FactorA=as.factor(FactorA)

FactorB=tabOrSpe[match(rownames(tabAggSimPerm),tabOrSpe$Sample),10]

FactorB=as.factor(FactorB)

FactorC=tabOrSpe[match(rownames(tabAggSimPerm),tabOrSpe$Sample),11]

FactorC=as.factor(FactorC)

#First permanova to obtain denominator SS and df

FirstPermanova=adonis(tabAggSimPerm~FactorA+FactorB%in%FactorA+FactorC%in%FactorB%in%FactorA,permutations=3,method="bray",strata=NULL)

mydf=FirstPermanova$aov.tab[2,1]

mySS=FirstPermanova$aov.tab[2,2]

#Second permanova with appropriate denominator

SecondPermanova=myAdonis(tabAggSimPerm~FactorA+FactorB%in%FactorA+FactorC%in%FactorB%in%FactorA,permutations=2000,method="bray",strata=FactorC%in%FactorB%in%FactorA,mySS.Res=mySS,mydf.Res=mydf,myIndex=3)

PermanovaRes=SecondPermanova$aov.tab[1,6]

vecAggPermSim=c(vecAggPermSim,PermanovaRes)

print(c(i,j))

}

#Put correlations in corresponding table assignement group

Data.res[,i]=vecAggCorSim

Data.res[,length(Data.groups)+i]=vecAggPermSim

}

################################################################################

#Step 2.3c Calculating correlations and PERMANOVA 2 CROSSED FACTORS #

################################################################################

#

#Reading original table

tabOrSpe=read.table("Data.o.csv",sep=";",header=T,strip.white=T,na.strings="NA",quote="")

#Obtaining the original triangular matrix at the species level

tabSpe=cast(tabOrSpe[,c(1:8)],Species+Genus+Family+Order+Class+Phylum~Sample)

tabSpeTri=vegdist(t(tabSpe[,c(7:ncol(tabSpe))]),method="bray")

#Creating empty table with correlations and PERMANOVA results

Data.res=as.data.frame(matrix(data=NA,nrow=1000,ncol=length(Data.groups)*2))

names(Data.res)=c(paste(as.character(Data.groups),"cor",sep="."),paste(as.character(Data.groups),"aov",sep="."))

#Loop on each aggregation levels

for (i in 1:length(Data.groups))

{

#Number of aggregation levels

AggNb=Data.groups[i]

AggIndex=seq(from=1,to=AggNb,by=1)

#Random assignments of simulated assignements on Species

vecAggCorSim=numeric(0)

vecAggPermSim=numeric(0)

for (j in 1:1000)

{

AggSim=sample(AggIndex,length(AggIndex),replace=F)

AggSimTemp=sample(AggIndex,nrow(tabSpe)-length(AggIndex),replace=T)

AggSim=c(AggSim,AggSimTemp)

rm(AggSimTemp)

AggSim=sample(AggSim,length(AggSim),replace=F)

tabSpeSim=cbind(AggSim,tabSpe)

tabAggSim=aggregate(tabSpeSim[,c(8:ncol(tabSpeSim))],by=list(tabSpeSim$AggSim),sum)

#Correlations between simulated assigments and original species

tabAggSimTriang=vegdist(t(tabAggSim[,c(2:ncol(tabAggSim))]),method="bray")

tabAggCorSim=cor(tabSpeTri,tabAggSimTriang,method="spearman")

vecAggCorSim=c(vecAggCorSim,tabAggCorSim)

#Permanova for each simulated assignement

#Creating the right format of the aggregated simulated matrix for permanova

tabAggSimPerm=t(tabAggSim[,c(2:ncol(tabAggSim))])

FactorA=tabOrSpe[match(rownames(tabAggSimPerm),tabOrSpe$Sample),9]

FactorA=as.factor(FactorA)

FactorB=tabOrSpe[match(rownames(tabAggSimPerm),tabOrSpe$Sample),10]

FactorB=as.factor(FactorB)

######### Step 2.3c.1. Term of interest: Factor A [FIXED] ###########

#First permanova to obtain denominator SS and df

FirstPermanova=adonis(tabAggSimPerm~FactorA+FactorB+FactorA*FactorB,permutations=3,method="bray",strata=NULL)

mydf=FirstPermanova$aov.tab[4,1]

mySS=FirstPermanova$aov.tab[4,2]

#Second permanova with appropriate denominator

SecondPermanova=myAdonis(tabAggSimPerm~FactorA+FactorB+FactorA*FactorB,permutations=2000,method="bray",strata=NULL,mySS.Res=mySS,mydf.Res=mydf,myIndex=3)

PermanovaRes=SecondPermanova$aov.tab[1,6]

vecAggPermSim=c(vecAggPermSim,PermanovaRes)

print(c(i,j))

}

#Put correlations in corresponding table assignement group

Data.res[,i]=vecAggCorSim

Data.res[,length(Data.groups)+i]=vecAggPermSim

}

######### Step 2.3c.2. Term of interest: Factor A [RANDOM] ###########

#First permanova to obtain denominator SS and df

FirstPermanova=adonis(tabAggSimPerm~FactorA+FactorB+FactorA*FactorB,permutations=3,method="bray",strata=NULL)

mydf=FirstPermanova$aov.tab[3,1]

mySS=FirstPermanova$aov.tab[3,2]

#Second permanova with appropriate denominator

SecondPermanova=myAdonis(tabAggSimPerm~FactorA+FactorB+FactorA*FactorB,permutations=2000,method="bray",strata=NULL,mySS.Res=mySS,mydf.Res=mydf,myIndex=3)

PermanovaRes=SecondPermanova$aov.tab[1,6]

vecAggPermSim=c(vecAggPermSim,PermanovaRes)

print(c(i,j))

}

#Put correlations in corresponding table assignement group

Data.res[,i]=vecAggCorSim

Data.res[,length(Data.groups)+i]=vecAggPermSim

}

######### Step 2.3c.3. Term of interest: Interaction A x B ###########

#First permanova to obtain denominator SS and df

FirstPermanova=adonis(tabAggSimPerm~FactorA+FactorB+FactorA*FactorB,permutations=3,method="bray",strata=NULL)

mydf=FirstPermanova$aov.tab[4,1]

mySS=FirstPermanova$aov.tab[4,2]

#Second permanova with appropriate denominator

SecondPermanova=myAdonis(tabAggSimPerm~FactorA+FactorB+FactorA*FactorB,permutations=2000,method="bray",strata=NULL,mySS.Res=mySS,mydf.Res=mydf,myIndex=3)

PermanovaRes=SecondPermanova$aov.tab[3,6]

vecAggPermSim=c(vecAggPermSim,PermanovaRes)

print(c(i,j))

}

#Put correlations in corresponding table assignement group

Data.res[,i]=vecAggCorSim

Data.res[,length(Data.groups)+i]=vecAggPermSim

}

##################################################################################

#Step 2.3d Calculating correlations and PERMANOVA 3 FACTORS [2 CROSSED 1 NESTED] #

##################################################################################

#Reading original table

tabOrSpe=read.table("Data.o.csv",sep=";",header=T,strip.white=T,na.strings="NA",quote="")

#Obtaining the original triangular matrix at the species level

tabSpe=cast(tabOrSpe[,c(1:8)],Species+Genus+Family+Order+Class+Phylum~Sample)

tabSpeTri=vegdist(t(tabSpe[,c(7:ncol(tabSpe))]),method="bray")

#Creating empty table with correlations and PERMANOVA results

Data.res=as.data.frame(matrix(data=NA,nrow=1000,ncol=length(Data.groups)*2))

names(Data.res)=c(paste(as.character(Data.groups),"cor",sep="."),paste(as.character(Data.groups),"aov",sep="."))

#Loop on each aggregation levels

for (i in 1:length(Data.groups))

{

#Number of aggregation levels

AggNb=Data.groups[i]

AggIndex=seq(from=1,to=AggNb,by=1)

#Random assignments of simulated assignements on Species

vecAggCorSim=numeric(0)

vecAggPermSim=numeric(0)

for (j in 1:1000)

{

AggSim=sample(AggIndex,length(AggIndex),replace=F)

AggSimTemp=sample(AggIndex,nrow(tabSpe)-length(AggIndex),replace=T)

AggSim=c(AggSim,AggSimTemp)

rm(AggSimTemp)

AggSim=sample(AggSim,length(AggSim),replace=F)

tabSpeSim=cbind(AggSim,tabSpe)

tabAggSim=aggregate(tabSpeSim[,c(8:ncol(tabSpeSim))],by=list(tabSpeSim$AggSim),sum)

#Correlations between simulated assigments and original species

tabAggSimTriang=vegdist(t(tabAggSim[,c(2:ncol(tabAggSim))]),method="bray")

tabAggCorSim=cor(tabSpeTri,tabAggSimTriang,method="spearman")

vecAggCorSim=c(vecAggCorSim,tabAggCorSim)

#Permanova for each simulated assignement

#Creating the right format of the aggregated simulated matrix for permanova

tabAggSimPerm=t(tabAggSim[,c(2:ncol(tabAggSim))])

FactorA=tabOrSpe[match(rownames(tabAggSimPerm),tabOrSpe$Sample),9]

FactorA=as.factor(FactorA)

FactorB=tabOrSpe[match(rownames(tabAggSimPerm),tabOrSpe$Sample),10]

FactorB=as.factor(FactorB)

FactorC=tabOrSpe[match(rownames(tabAggSimPerm),tabOrSpe$Sample),11]

FactorC=as.factor(FactorC)

######### Step 2.3d.1. Term of interest: Factor A ###########

#First permanova to obtain denominator SS and df

FirstPermanova=adonis(tabAggSimPerm~FactorA+FactorB+FactorC%in%FactorB+FactorA*FactorB+FactorA*FactorC%in%FactorB,permutations=3,method="bray",strata=NULL)

mydf=FirstPermanova$aov.tab[4,1]

mySS=FirstPermanova$aov.tab[4,2]

#Second permanova with appropriate denominator

SecondPermanova=myAdonis(tabAggSimPerm~FactorA+FactorB+FactorC%in%FactorB+FactorA*FactorB+FactorA*FactorC%in%FactorB,permutations=2000,method="bray",strata=FactorC%in%FactorB,mySS.Res=mySS,mydf.Res=mydf,myIndex=3)

#Put correlations in corresponding table assignement group

Data.res[,i]=vecAggCorSim

Data.res[,length(Data.groups)+i]=vecAggPermSim

}

######### Step 2.3d.2. Term of interest: Interaction A x B ###########

#First permanova to obtain denominator SS and df

FirstPermanova=adonis(tabAggSimPerm~FactorA+FactorB+FactorC%in%FactorB+FactorA*FactorB+FactorA*FactorC%in%FactorB,permutations=3,method="bray",strata=NULL)

mydf=FirstPermanova$aov.tab[5,1]

mySS=FirstPermanova$aov.tab[5,2]

#Second permanova with appropriate denominator

SecondPermanova=myAdonis(tabAggSimPerm~FactorA+FactorB+FactorC%in%FactorB+FactorA*FactorB+FactorA*FactorC%in%FactorB,permutations=2000,method="bray",strata=FactorC%in%FactorB,mySS.Res=mySS,mydf.Res=mydf,myIndex=4)

PermanovaRes=SecondPermanova$aov.tab[4,6]

vecAggPermSim=c(vecAggPermSim,PermanovaRes)

print(c(i,j))

}

#Put correlations in corresponding table assignement group

Data.res[,i]=vecAggCorSim

Data.res[,length(Data.groups)+i]=vecAggPermSim

}

################################

#Step 2.4 Writing result table #

################################

#

write.table(Data.res,"Data.res.csv",sep=";",row.names=F,col.names=T,quote=F)

################################################################################

# 3. Loss of information between original vs. taxonomically #

# aggregated matrices and comparison with random expectations #

################################################################################

#

###############################################################################

# Step.3.1 Reading the data and creating the table of info on original groups #

###############################################################################

#

#Dataframe of groups informations

tabinfo=as.data.frame(matrix(data=NA,nrow=1,ncol=6))

names(tabinfo)=c("Species","Genus","Family","Order","Class","Phylum")

#Dataframe of Original and Mean and CI simluated corelations

tabCI=as.data.frame(matrix(data=NA,nrow=3,ncol=6))

names(tabCI)=c("Type","Genus","Family","Order","Class","Phylum")

tabCI$Type=rep(c("CorOr","CorSim","CorSimCI"),1)

#Number of groups (G) for each dataset and for each taxon

for (i in 1:nrow(tabinfo))

{

tab=read.table("Data.o.csv",sep=";",header=T,strip.white=T,na.strings="NA",quote="")

tabinfo[i,"Species"]=length(unique(tab$Species))

tabinfo[i,"Genus"]=length(unique(tab$Genus))

tabinfo[i,"Family"]=length(unique(tab$Family))

tabinfo[i,"Order"]=length(unique(tab$Order))

tabinfo[i,"Class"]=length(unique(tab$Class))

tabinfo[i,"Phylum"]=length(unique(tab$Phylum))

}

rm(tab)

#

################################################################################

# Step.3.2 Calculating correlations between original tables with original #

# groups and simulated groups #

################################################################################

#

#Loading requested libraries and functions

library(reshape)

library(vegan)

myAggregate=function(myX, myBy, myFun, myCollapse=':', ...)

{

myNames=colnames(myBy)

myBy=apply(myBy, 1, paste, collapse=myCollapse)

myRes=aggregate(myX, list(myBy), myFun, ...)

myRes=cbind(as.data.frame(matrix(unlist(strsplit(as.character(myRes[,1]), myCollapse)),ncol=length(myNames), byrow=T)), myRes[,-1])

names(myRes)[1:length(myNames)]=myNames

myRes

}

#Empty list for correlation results

listinfo=list()

#Calculating original and simulated correlations for each taxonomic level

#Reading original table

tabOrSpe=read.table("Data.o.csv",sep=";",header=T,strip.white=T,na.strings="NA",quote="")

#Removing sampling factors

tabOrSpe=tabOrSpe[,c(1:8)]

#Obtaining the original triangular matrix at the species level

tabSpe=cast(tabOrSpe,Species+Genus+Family+Order+Class+Phylum~Sample)

tabSpeTri=vegdist(t(tabSpe[,c(7:ncol(tabSpe))]),method="bray")

#

##########################

# Step.3.2.1 Genus Level #

##########################

#

#

#Step 3.2.1.1 --- Original correlation

#

tabGen=myAggregate(tabOrSpe$Ab,tabOrSpe[,c(2,7)],sum,myCollapse=':',na.rm=T)

names(tabGen)[length(tabGen)]='Ab'

tabGen=cast(tabGen,Genus~Sample)

tabGen=vegdist(t(tabGen[,c(2:ncol(tabGen))]),method="bray")

tabGenCorOr=cor(tabSpeTri,tabGen,method="spearman")

listinfo=c(listinfo,temp=list(tabGenCorOr))

#Putting original correlation in tabCI

tabCI[tabCI$Type=="CorOr",2]=tabGenCorOr

#

#Step 3.2.1.2 --- Simulated correlations

#

#Number of Genus levels

GenNb=tabinfo[1,"Genus"]

GenIndex=seq(from=1,to=GenNb,by=1)

#Random assignments of simulated Genus on Species

vecGenCorSim=numeric(0)

for (i in 1:1000)

{

GenSim=sample(GenIndex,length(GenIndex),replace=F)

GenSimTemp=sample(GenIndex,nrow(tabSpe)-length(GenIndex),replace=T)

GenSim=c(GenSim,GenSimTemp)

rm(GenSimTemp)

GenSim=sample(GenSim,length(GenSim),replace=F)

tabSpeSim=cbind(GenSim,tabSpe)

#Correlations between simulated Genus and original species

tabGenSim=aggregate(tabSpeSim[,c(8:ncol(tabSpeSim))],by=list(tabSpeSim$GenSim),sum)

tabGenSim=vegdist(t(tabGenSim[,c(2:ncol(tabGenSim))]),method="bray")

tabGenCorSim=cor(tabSpeTri,tabGenSim,method="spearman")

vecGenCorSim=c(vecGenCorSim,tabGenCorSim)

}

#Vector of simulated correlations assigned to listinfo

listinfo=c(listinfo,temp=list(vecGenCorSim))

#Putting simulated mean and CI correlation in tabCI

tabCI[tabCI$Type=="CorSim",2]=mean(vecGenCorSim)

tabCI[tabCI$Type=="CorSimCI",2]=1.96*sqrt(var(vecGenCorSim))

rm(GenIndex,GenNb,GenSim,tabGen,tabGenCorOr,tabGenCorSim,tabGenSim,tabSpeSim,vecGenCorSim)

#

###########################

# Step.3.2.2 Family Level #

###########################

#

#

#Step 3.2.2.1 --- Original correlation

#

tabFam=myAggregate(tabOrSpe$Ab,tabOrSpe[,c(3,7)],sum,myCollapse=':',na.rm=T)

names(tabFam)[length(tabFam)]='Ab'

tabFam=cast(tabFam,Family~Sample)

tabFam=vegdist(t(tabFam[,c(2:ncol(tabFam))]),method="bray")

tabFamCorOr=cor(tabSpeTri,tabFam,method="spearman")

listinfo=c(listinfo,temp=list(tabFamCorOr))

#Putting original correlation in tabCI

tabCI[tabCI$Type=="CorOr",3]=tabFamCorOr

#

#Step 3.2.2.2 --- Simulated correlations

#

#Number of Family levels

FamNb=tabinfo[1,"Family"]

FamIndex=seq(from=1,to=FamNb,by=1)

#Random assignments of simulated Family on Species

vecFamCorSim=numeric(0)

for (i in 1:1000)

{

FamSim=sample(FamIndex,length(FamIndex),replace=F)

FamSimTemp=sample(FamIndex,nrow(tabSpe)-length(FamIndex),replace=T)

FamSim=c(FamSim,FamSimTemp)

rm(FamSimTemp)

FamSim=sample(FamSim,length(FamSim),replace=F)

tabSpeSim=cbind(FamSim,tabSpe)

#Correlations between simulated Family and original species

tabFamSim=aggregate(tabSpeSim[,c(8:ncol(tabSpeSim))],by=list(tabSpeSim$FamSim),sum)

tabFamSim=vegdist(t(tabFamSim[,c(2:ncol(tabFamSim))]),method="bray")

tabFamCorSim=cor(tabSpeTri,tabFamSim,method="spearman")

vecFamCorSim=c(vecFamCorSim,tabFamCorSim)

}

#Vector of simulated correlations assigned to listinfo

listinfo=c(listinfo,temp=list(vecFamCorSim))

#Putting simulated mean and CI correlation in tabCI

tabCI[tabCI$Type=="CorSim",3]=mean(vecFamCorSim)

tabCI[tabCI$Type=="CorSimCI",3]=1.96*sqrt(var(vecFamCorSim))

rm(FamIndex,FamNb,FamSim,tabFam,tabFamCorOr,tabFamCorSim,tabFamSim,tabSpeSim,vecFamCorSim)

#

##########################

# Step.3.2.3 Order Level #

##########################

#

#

#Step 3.2.3.1 --- Original correlation

#

tabOrd=myAggregate(tabOrSpe$Ab,tabOrSpe[,c(4,7)],sum,myCollapse=':',na.rm=T)

names(tabOrd)[length(tabOrd)]='Ab'

tabOrd=cast(tabOrd,Order~Sample)

tabOrd=vegdist(t(tabOrd[,c(2:ncol(tabOrd))]),method="bray")

tabOrdCorOr=cor(tabSpeTri,tabOrd,method="spearman")

listinfo=c(listinfo,temp=list(tabOrdCorOr))

#Putting original correlation in tabCI

tabCI[tabCI$Type=="CorOr",4]=tabOrdCorOr

#

#Step 3.2.3.2 --- Simulated correlations

#

#Number of Order levels

OrdNb=tabinfo[1,"Order"]

OrdIndex=seq(from=1,to=OrdNb,by=1)

#Random assignments of simulated Order on Species

vecOrdCorSim=numeric(0)

for (i in 1:1000)

{

OrdSim=sample(OrdIndex,length(OrdIndex),replace=F)

OrdSimTemp=sample(OrdIndex,nrow(tabSpe)-length(OrdIndex),replace=T)

OrdSim=c(OrdSim,OrdSimTemp)

rm(OrdSimTemp)

OrdSim=sample(OrdSim,length(OrdSim),replace=F)

tabSpeSim=cbind(OrdSim,tabSpe)

#Correlations between simulated Order and original species

tabOrdSim=aggregate(tabSpeSim[,c(8:ncol(tabSpeSim))],by=list(tabSpeSim$OrdSim),sum)

tabOrdSim=vegdist(t(tabOrdSim[,c(2:ncol(tabOrdSim))]),method="bray")

tabOrdCorSim=cor(tabSpeTri,tabOrdSim,method="spearman")

vecOrdCorSim=c(vecOrdCorSim,tabOrdCorSim)

}

#Vector of simulated correlations assigned to listinfo

listinfo=c(listinfo,temp=list(vecOrdCorSim))

#Putting simulated mean and CI correlation in tabCI

tabCI[tabCI$Type=="CorSim",4]=mean(vecOrdCorSim)

tabCI[tabCI$Type=="CorSimCI",4]=1.96*sqrt(var(vecOrdCorSim))

rm(OrdIndex,OrdNb,OrdSim,tabOrd,tabOrdCorOr,tabOrdCorSim,tabOrdSim,tabSpeSim,vecOrdCorSim)

#

##########################

# Step.3.2.4 Class Level #

##########################

#

#

#Step 3.2.4.1 --- Original correlation

#

tabCla=myAggregate(tabOrSpe$Ab,tabOrSpe[,c(5,7)],sum,myCollapse=':',na.rm=T)

names(tabCla)[length(tabCla)]='Ab'

tabCla=cast(tabCla,Class~Sample)

tabCla=vegdist(t(tabCla[,c(2:ncol(tabCla))]),method="bray")

tabClaCorOr=cor(tabSpeTri,tabCla,method="spearman")

listinfo=c(listinfo,temp=list(tabClaCorOr))

#Putting original correlation in tabCI

tabCI[tabCI$Type=="CorOr",5]=tabClaCorOr

#

#Step 3.2.4.2 --- Simulated correlations

#

#Number of Class levels

ClaNb=tabinfo[1,"Class"]

ClaIndex=seq(from=1,to=ClaNb,by=1)

#Random assignments of simulated Class on Species

vecClaCorSim=numeric(0)

for (i in 1:1000)

{

ClaSim=sample(ClaIndex,length(ClaIndex),replace=F)

ClaSimTemp=sample(ClaIndex,nrow(tabSpe)-length(ClaIndex),replace=T)

ClaSim=c(ClaSim,ClaSimTemp)

rm(ClaSimTemp)

ClaSim=sample(ClaSim,length(ClaSim),replace=F)

tabSpeSim=cbind(ClaSim,tabSpe)

#Correlations between simulated Class and original species

tabClaSim=aggregate(tabSpeSim[,c(8:ncol(tabSpeSim))],by=list(tabSpeSim$ClaSim),sum)

tabClaSim=vegdist(t(tabClaSim[,c(2:ncol(tabClaSim))]),method="bray")

tabClaCorSim=cor(tabSpeTri,tabClaSim,method="spearman")

vecClaCorSim=c(vecClaCorSim,tabClaCorSim)

}

#Vector of simulated correlations assigned to listinfo

listinfo=c(listinfo,temp=list(vecClaCorSim))

#Putting simulated mean and CI correlation in tabCI

tabCI[tabCI$Type=="CorSim",5]=mean(vecClaCorSim)

tabCI[tabCI$Type=="CorSimCI",5]=1.96*sqrt(var(vecClaCorSim))

rm(ClaIndex,ClaNb,ClaSim,tabCla,tabClaCorOr,tabClaCorSim,tabClaSim,tabSpeSim,vecClaCorSim)

#

###########################

# Step.3.2.5 Phylum Level #

###########################

#

#

#Step 3.2.5.1 --- Original correlation

#

tabPhyl=myAggregate(tabOrSpe$Ab,tabOrSpe[,c(6,7)],sum,myCollapse=':',na.rm=T)

names(tabPhyl)[length(tabPhyl)]='Ab'

tabPhyl=cast(tabPhyl,Phylum~Sample)

tabPhyl=vegdist(t(tabPhyl[,c(2:ncol(tabPhyl))]),method="bray")

tabPhylCorOr=cor(tabSpeTri,tabPhyl,method="spearman")

listinfo=c(listinfo,temp=list(tabPhylCorOr))

#Putting original correlation in tabCI

tabCI[tabCI$Type=="CorOr",6]=tabPhylCorOr

#

#Step 3.2.5.2 --- Simulated correlations

#

#Number of Phylum levels

PhyNb=tabinfo[1,"Phylum"]

PhyIndex=seq(from=1,to=PhyNb,by=1)

#Random assignments of simulated Class on Species

vecPhyCorSim=numeric(0)

for (i in 1:1000)

{

PhySim=sample(PhyIndex,length(PhyIndex),replace=F)

PhySimTemp=sample(PhyIndex,nrow(tabSpe)-length(PhyIndex),replace=T)

PhySim=c(PhySim,PhySimTemp)

rm(PhySimTemp)

PhySim=sample(PhySim,length(PhySim),replace=F)

tabSpeSim=cbind(PhySim,tabSpe)

#Correlations between simulated Phylum and original species

tabPhySim=aggregate(tabSpeSim[,c(8:ncol(tabSpeSim))],by=list(tabSpeSim$PhySim),sum)

tabPhySim=vegdist(t(tabPhySim[,c(2:ncol(tabPhySim))]),method="bray")

tabPhyCorSim=cor(tabSpeTri,tabPhySim,method="spearman")

vecPhyCorSim=c(vecPhyCorSim,tabPhyCorSim)

}

#Vector of simulated correlations assigned to listinfo

listinfo=c(listinfo,temp=list(vecPhyCorSim))

#Putting simulated mean and CI correlation in tabCI

tabCI[tabCI$Type=="CorSim",6]=mean(vecPhyCorSim)

tabCI[tabCI$Type=="CorSimCI",6]=1.96*sqrt(var(vecPhyCorSim))

rm(PhyIndex,PhyNb,PhySim,tabPhyl,tabPhylCorOr,tabPhyCorSim,tabPhySim,tabSpeSim,vecPhyCorSim)

###############################################################################

# Step.3.3 Writing table of original and simulated correlations #

# and CI for each taxonomic level #

###############################################################################

#

write.table(tabCI,"tax.corr.res.csv",sep=";",row.names=F,col.names=T,quote=F)

**Appendix S2. User guide to the R code.**

The R code for BestAgg analyses (Appendix S1) is articulated in 3 main procedures:

1. *Creating the database format from Species × Sample matrix*

Implementation of a database, which is necessary to perform analyses in procedures 2 and 3, starting from the original species × sample data matrix.

1. *Calculating simulated correlation and PERMANOVA results for each group of random surrogates*

This procedure allows identifying the minimum number of surrogate groups *G*_min_ sufficient to obtain results consistent with those obtained at species level, quantifying the information retained in the BestAgg aggregated matrix, and defining the probability of type I error when using *G*_BestAgg_ as the effective number of surrogates (see Methods for further details).

1. *Loss of information between original vs. taxonomically aggregated matrices and comparison with random expectations*

Randomization test on classic taxonomic surrogates allowing checking for the amount of information retained in matrix aggregated following the Linnaean hierarchy.

Note that **procedures 2 and 3 are independent but require necessarily the database format provided by procedure 1**. Note also that **procedure 2** (Appendix S1) **is provided for most common experimental designs with 2 or 3 factors,** whereas more complex designs require some modifications of the R code.

Multivariate statistical tests in the R code are based by default on **PERMANOVA**. PERMANOVA allows analyzing complex multi-factorial designs, testing for interaction terms, using any distance measure, and does not assume normal distribution of data (see text for further details). However, the R code is flexible to changes in the type of multivariate test, if necessary.

The R code has been thought to identify the lowest number of surrogates (*G*_min_) sufficient for analyses in experimental contexts where the experimenter aims at assessing if a given factor of interest determines significant change in a given community. Clearly the same logic of BestAgg could be extended also to experimental contexts where the interest lies in determining correlations between spatio-temporal patterns in communities and environmental variables (whether categorical or continuous), although this would require the identification of *G*_min_ to be based on descriptors other than *P*-values from multivariate analyses, such as *R^2^* values, for instance, in correlation studies.

**1. Create the database format from Species × Sample matrix**

**This first procedure** allows constructing the required data format, which **is necessary for subsequent analyses** (procedures 2 and 3). **Two input files (.csv)** are needed for this step. **A first file contains the Species × Sample data matrix**, in which species are rows and sample are columns. For each species, the matrix should specify also the full taxonomic tree, from species to phylum (see the example data “Data.csv” in Appendix S3). Species’ authors are not necessary. **The second file contains factors for PERMANOVA analyses** (see the example file “Factors.csv” in Appendix S4).

The input file for data must be named “**Data.csv**”. Note that **columns involving taxonomic information in “Data.csv” must be named as “Species”, “Genus”, Family”, “Order”, “Class”, “Phylum” and provided in this precise order**. If variables are all species and interest lies also in checking for the information retained at higher taxonomic resolution using procedure 3, the full taxonomic hierarchy must be provided as in the following example:

Species Genus Family Order Class Phylum

*C. ionica Cocculina* Cocculinidae Cocculiniformia Gastropoda Mollusca

If variables are not all species but interest still lies also in checking for the information retained at higher taxonomic resolution, the full taxonomic hierarchy must be provided as above for species, and as in the following examples for other variables (e.g. higher taxa, morphological groups, etc.):

Species Genus Family Order Class Phylum

Paraonidae Paraonidae Paraonidae Scolecida Polychaeta Annelida

Erect sponges Erect sponges Erect sponges Erect sponges Erect sponges Porifera

If procedure 3 is not in the aims of the investigator, irrespective of variables types, columns for the taxonomic hierarchy could be filled as follows. For instance:

Species Genus Family Order Class Phylum

*C. ionica C. ionica C. ionica* *C. ionica* *C. ionica* *C. ionica*

Paraonidae Paraonidae Paraonidae Paraonidae Paraonidae Paraonidae

Erect sponges Erect sponges Erect sponges Erect sponges Erect sponge Erect sponges

The input file for factors must be named “**Factors.csv**”. **Columns in “Factors.csv” must be named “FactorA”, “FactorB”, and “FactorC”**. **Column for Factor C must be provided even when analyzing 2-factor designs, and filled with all “0” values** (see the example file “Factors.csv” in Appendix S4).

Note that **the sequence of factor levels in “Factors.csv” has to correspond to the sequence of samples in “Data.csv”**. Check carefully input files for errors, symbols not recognized by R, and so on. Input files must be exactly in the form of example files (see Appendices S3-S4), with names and numbers separated by semicolons.

**The output of the analysis is a .csv file named by default “Data.o.csv”**, containing the database required for subsequent analyses.

**2. Calculating simulated correlation and PERMANOVA results for each group of random surrogates**

This part of the R code contains the script calculating (a) correlation ρ values between the species-level data matrix and randomly aggregated matrices for each *G* obtained from the step-wise reduction of fixed detriments *d*, and (b) PERMANOVA results for each randomly aggregated matrix. This procedure also allows calculating the correlation ρ values between the species-level matrix and matrices in which species are randomly aggregated in the *G*_BestAgg_ groups (see Methods, see also Appendix S5), in order to check the amount of information on species-level community patterns retained and the probability of type I error when using *G*_BestAgg_ surrogates.

The first step here (*Step 2.1 Build sub-groups table – Determine number of sub-groups for each dataset*) serves to define the set of *G* groups in which species will be aggregated at random. *G* groups come from the step-wise reduction of the original number of species *S*. *S* is progressively reduced by fixed detriments *d* = 10% *S*, or if *S* is very high, by *d* = 5% *S* (see Methods). The line of the script:

(1) *Data.groups=c(247,234,221,208,195,182,169,156,143,130,117,104,91,78,65,52,39,26,13)*

contains, at the present, the *G* groups defined for the example data provided in Appendix S3, which refers to the to the platform P1 of the OP case study. In this case *S* = 259 and a fixed detriment of *d* = 5% *S* = 13 (approximated) was applied. Considering approximately 260 species and detriments of 13, the original species were randomly aggregated in 247, 234, 221,…, 39, 26, 13 groups. Therefore, **in this line of the R code, the sequence of *G* groups in which species have to be aggregated based on chosen detriments should be inserted in brackets**. The R code performs **1,000 random aggregations** **for each *G***.

The next step (*Step 2.2 Loading required functions*) involves **loading libraries and/or functions for analyses**.

**Subsequent steps**, instead, include scripts **performing PERMANOVA analyses and calculating ρ values**:

1. *Step 2.3a Calculating correlations and PERMANOVA 2 NESTED FACTORS*. It applies to **nested designs involving 2 factors**. In this case, **factor A may be fixed or random** **and** **factor** **B** **is** **random and** **nested in A** and the **term of interest** for the analysis **is A**. Example 1: Location, random, *n* levels (Factor A); Site, random, nested in Location, *m* levels (Factor B). Example 2 (as in the OP case study): Distance from impact, fixed, *n* levels (Factor A); Site, random, nested in Distance, *m* levels (Factor B).
2. *Step 2.3b Calculating correlations and PERMANOVA 3 NESTED FACTORS*. It applies to **nested designs involving 3 factors**. In this case, **factor A may be fixed or random, factor** **B** **is** **random and** **nested in A, and factor C is random and nested in B**. The **term of interest** for the analysis **is A**. Example 1: Location, random, *n* levels (Factor A); Site, random, nested in Location, *m* levels (Factor B); Plot, random, nested in Site, *q* levels. Example 2: Impact, fixed, 2 (Impact and Control) levels (Factor A); Location, random, nested in Impact, *n* levels (Factor B); Site, random, nested in Location, *m* levels (Factor C).
3. *Step 2.3c Calculating correlations and PERMANOVA 2 CROSSED FACTORS*. It applies to **designs involving 2 crossed factors**. In this case, **A and B may be fixed or random**.
   1. The sub-step 1 (*Step 2.3c.1. Term of interest: Factor A [FIXED]*) applies when **Factor A is fixed and is the term of interest for the analysis**, **Factor B may be fixed or random**. Example 1: Habitat, fixed, *n* levels (Factor A); Season, fixed, *m* levels (Factor B). Example 2: Impact, fixed, 2 (Impact and Control) levels (Factor A); Time, random, *m* levels (Factor B).
   2. The sub-step 2 (*Step 2.3c.2. Term of interest: Factor A [RANDOM]*) applies when **Factor A is random and is the term of interest for the analysis**, **Factor B is random**. Example: Time, random, *n* levels (Factor A); Location, random, *m* levels (Factor B).
   3. The sub-step 3 (*Step 2.3c.3. Term of interest: Interaction A × B*) applies when **the term of interest for the analysis is the interaction term Factor A × Factor B**, **whether Factor A and B are either fixed or random**.
4. *Step 2.3d Calculating correlations and PERMANOVA 3 FACTORS [2 CROSSED 1 NESTED]* applies to **designs involving 3 crossed and nested factors**. In this case, **Factor** **A is fixed, Factor B is random, and Factor C is random and nested in Factor B**. Example (as in the DG case study): Depth, fixed, *n* levels (Factor A); Location, random, *m* level (Factor B); Site, random, nested in Location, *q* levels (Factor C).
   1. The sub-step 1 (*Step 2.3d.1. Term of interest: Factor A*) applies when **Factor A is the term of interest for the analysis**.
   2. The sub-step 2 (*Step 2.3d.2. Term of interest: Interaction A × B*) applies when **the term of interest for the analysis is the interaction term Factor A × Factor B**.

Note that **PERMANOVA analyses are set, by default, on Bray-Curtis dissimilarities of untransformed data, with 2,000 permutations**. However, PERMANOVA analyses may be done using any distance metric and transformation of data after modifying the R code appropriately. Note that, depending on the size of the dataset, the number of step-wise reductions, i.e. numbers in line (1) (see above), and number of permutations used for PERMANOVA, analyses may have different duration, from hours to days. In this view, the number of permutations for PERMANOVA has been fixed to 2,000, allowing testing terms of interest even with *P <* 0.001. More conservative significance levels may be obtained increasing the number of permutations, although the time required for analyses may rise consequently.

**The final step** of this procedure (*Step 2.4 Writing result table*) **provides** **a .csv file**, named by default as “**Data.res.csv**” **containing results** of previous analyses. In the result table, **columns reporting correlation** **ρ values from 1,000 random aggregations for each *G*** **are** **indicated with** **the number of the specific *G* groups** **followed by “.cor”** (e.g. “247.cor”), whereas the remaining **columns reporting** ***P-*values of the test of interest from the corresponding 1,000 PERMANOVA analyses are indicated with the number of the specific *G* groups followed by “.aov”** (e.g. “247.aov”).

Based on such results, the % of analyses for each *G* giving consistent results with those obtained analyzing species-level data can be easily calculated, and regression analyses of ρ against ln(φ) (where φ = *G*/*S*, see Methods) can be then performed using any statistical package. **The lowest *G* allowing at least 95% of PERMANOVA analyses to give a *P*-value equal or lower than the significance level obtained analyzing species-level data represents the sufficient number of surrogate groups *G*_min_** (see Methods for further details). Note that the procedure allows identifying *G*_min_ directly, whereas **the corresponding sufficient level of aggregation is given as φ_low_ = *G*_min_/*S***.

Once *G*_min_ has been defined, surrogates for BestAgg can be selected, and the final number of BestAgg surrogates, namely *G*_BestAgg_, obtained (see Appendix S5, see also Methods). The above procedure also allows **checking for the amount of information on species-level community patterns retained when using BestAgg surrogates**, **expressed as ρ_BestAgg_** (i.e. the correlation between the original species-level matrix and the matrix in which species have been aggregated in the BestAgg surrogates, which is not provided by the R code but can be calculated using any statistical computer program), and **defining the probability of type I error for *G*_BestAgg_**. The whole procedure, in this case, can be performed **inserting the number of *G*_BestAgg_ in the line (1) of the R code**. For instance, for the example data provided in Appendix S3 (referred to platform P1 of OP case study), *G*_min_ = 26 and the selection procedure led to *G*_BestAgg_ = 29 (see Table S3). Thus, the line (1) will be:

(2) *Data.groups=c(29)*

At end of the analysis, **in this case, the resulting file “Data.res.csv” will report correlation ρ values and *P*-values specific for *G*_BestAgg_**. The **ρ values obtained in this way can be used to construct the 95%CI or frequency distribution against which testing ρ_BestAgg_** (e.g. see Figure 2). As previously described, **the % of PERMANOVA analyses showing *P*-values equal or lower than the significance level of species-level analyses represents the probability of type I error specific for *G*_BestAgg_** (under the null hypothesis that BestAgg surrogates are random subsets of the original pool of species).

In this framework, any set of surrogates (e.g. using families as surrogates of species) can be checked. Just to insert in line (1) of the R code the number of surrogates of the set to obtain ρ values and *P*-values from 1,000 randomizations, which can be then used, as explained above, to the check the amount of information retained and define the related probability of type I error.

**3. Loss of information between original vs. taxonomically aggregated matrices and comparison with random expectations**

This procedure allows **calculating correlation ρ values between the species-level matrix and matrices aggregated using classic taxonomic surrogates** (i.e. matrices in which species are aggregated in taxa of higher taxonomic rank, such as Genus, Family, Order, Class, and Phylum), **and correlation ρ values between the species-level matrix and matrices in which species are randomly aggregated in classic taxonomic surrogates** (1,000 random aggregations for each taxonomic rank), in order **to check for the information contained in taxonomically aggregated matrices**. The final step of the procedure (*Step.3.3 Writing table of original and simulated correlations and CI for each taxonomic level*) provides a .csv file, named by default as “**tax.corr.res.csv**” containing ρ values between species vs. higher-taxon matrices (indicated as “CorOr” in the result table), mean ρ values between species vs. randomly aggregated matrices ± 95%CI from 1,000 random aggregations for each taxonomic rank (indicated in the result table as “CorSim” and “CorSimCI” respectively).

**Appendix S3.** Example data: species level matrix of P1 (OP case study). Provided as separate .csv file (see SupplInfo_AppendixS3_example_data.csv).

**Appendix S4.** Factors for example data. Provided as separate .csv file (see SupplInfo_AppendixS4_factors.csv).
